# Effects of vitamin and multiple micronutrient supplementation for pregnant and/or lactating women on maternal and infant nutritional status in low- and middle-income countries: a systematic review and meta-analysis

**DOI:** 10.1016/j.advnut.2025.100487

**Published:** 2025-08-05

**Authors:** Sachin Shinde, Cara A Yelverton, Mashavu Yussuf, Lina Nurhussien, Dongqing Wang, Wafaie W Fawzi

**Affiliations:** 1 Department of Global Health and Population, Harvard T. H. Chan School of Public Health, Boston, Massachusetts, United States of America; 2 Center for Inquiry into Mental Health, Pune, India; 3 Africa Academy for Public Health, Dar Salaam, Tanzania; 4 Department of Global and Community Health, College of Public Health, George Mason University, Fairfax, Virginia, United States of America; 5 Department of Epidemiology, Harvard T. H. Chan School of Public Health, Boston, Massachusetts, United States of America; 6 Department of Nutrition, Harvard T. H. Chan School of Public Health, Boston, Massachusetts, United States of America

**Keywords:** antenatal care, postnatal supplementation, maternal supplementation, micronutrient deficiencies, nutritional status, randomized controlled trials, evidence synthesis, low- and middle-income countries

## Abstract

**Background:**

Globally, over half of women of reproductive age are affected by at least one micronutrient deficiency, often exacerbated during pregnancy and lactation, contributing to adverse maternal and child health outcomes. This systematic review and meta-analysis synthesized impact of vitamin supplementation on maternal, infant and lactational nutritional status in low- and middle-income countries.

**Methods:**

MEDLINE, EMBASE, CENTRAL, and WHO library databases were searched. Eligible studies included randomized controlled trials of micronutrient supplementation in healthy pregnant/lactating women, assessing maternal/infant micronutrient status or milk composition. Random-effects meta-analysis was performed for outcomes with ≥2 studies, and evidence quality was evaluated using GRADE.

**Results:**

Eighty-seven papers (76 trials, including 65 for meta-analysis) were included. Maternal vitamin B-12 supplementation during pregnancy increased serum cobalamin concentrations (standard mean difference [SMD] 0.39; 95% CI 0.11, 0.68; *P*=0.01) and reduced deficiency (OR 0.43; 95% CI 0.19, 0.95; *P*=0.04), with improved B-12 concentrations in milk, especially when administered postpartum (SMD 0.33; 95% CI 0.02, 0.63; P=0.04), but had no consistent effect on infant or cord serum cobalamin concentrations. Vitamin A supplementation during pregnancy or postpartum improved maternal serum concentrations (SMD 0.60; 95% CI 0.13, 1.08; *P*<0.001) and reduced deficiency at thresholds ≤0.7 μmol/L (OR 0.55; 95% CI 0.43, 0.71; *P*<0.001); however, its effects on infant and cord serum levels were negligible. Postpartum vitamin A supplementation improved milk vitamin A concentrations (SMD 0.53; 95% CI 0.19, 0.86; *P*<0.001), particularly with single high-dose regimens. Supplementation with vitamin D during pregnancy increased maternal serum vitamin D concentrations (SMD 1.68; 95% CI 0.99, 2.37; *P*<0.001), reduced deficiency at thresholds ≤50 nmol/L (OR 0.30; 95% CI 0.14, 0.64; *P*<0.001) and increased vitamin D concentrations in infant and cord serum.

**Conclusions:**

Micronutrient supplementation during pregnancy and lactation improved maternal nutritional status but showed inconsistent effects on infant nutritional status, highlighting the need for further research.

**PROSPERO Registration ID:**

CRD42022308715; https://tinyurl.com/y33cxekr.

**Statement of Significance**

This review consolidates fragmented evidence on vitamin supplementation in LMICs and provides actionable insights for program design and policy, aiming to improve maternal and infant nutrition.

## Introduction

Globally, more than half of women of reproductive age (15-49 years) in low- and middle-income countries (LMICs) are affected by at least one micronutrient deficiency, with particularly high burdens in South Asia and sub-Saharan Africa [1]. Women in these countries commonly experience multiple micronutrient deficiencies due to inadequate intake of nutrient-dense foods such as fruits, vegetables, animal source foods, and fortified foods, but also because of dietary patterns rich in components like phytates, oxalates, and polyphenols [2]. These substances, especially prevalent in rural diets, can inhibit the absorption of key nutrients including iron, zinc, and calcium, thereby contributing to deficiencies [1]. Chronic diseases and infections further impair nutrient absorption [3]. Pregnancy and lactation exacerbate deficiencies due to increased demands, often worsened by repeaed pregnancies and short birth intervals [4,5]. These deficiencies threaten maternal and infant health and perpetuate intergenerational malnutrition [4,6,7].

Micronutrient deficiencies, particularly folate, iron, and iodine, are well-documented contributors to abnormal prenatal development and/or adverse pregnancy outcomes. Less recognized are deficiencies in B-vitamins (elevating homocysteine) and vitamin D, linked to neural tube defects, preeclampsia, preterm birth, and perinatal death [8]. Maternal antioxidant status, influenced by various factors including vitamins C and E, also plays a crucial role in preventing pregnancy complications [4]. Maternal nutrition also influences infant immune system, cardiovascular system, brain and cognitive development [9], while lactation depletes maternal reserves, with limited research on its effects on maternal health.

Strategies to address micronutrient deficiencies include diversified diets, food fortification, biofortification, and supplementation [1,10]. While the World Health Organization (WHO) recommends daily iron and folic acid (IFA) supplementation during pregnancy, recent studies support multiple micronutrient supplements (MMS) to reduce risks of low birth weight, small-for-gestational-age births, and preterm births [1,11]. However, the WHO has not yet recommended MMS as an alternative to prenatal IFA and advises MMS only in the context of rigorous research [12]. Therefore, assessing the content and effect of maternal supplements is crucial to ensure that pregnant women receive the appropriate nutrients, minimize deficiency risks, and improve maternal-infant health effectively in LMICs [4,13]. Given the extended nutritional demands of lactation, supplementing breastfeeding mothers with MMS could effectively enhance maternal and infant nutrition and health [1,4,12].

Historically, maternal and infant nutrition research focused on individual micronutrients like iron, folate, and vitamin A due to their well-documented effects on maternal and child health [1,4,12]. Recently, interest has shifted to MMS during pregnancy and lactation, though uncertainties remain about optimal micronutrient composition, dosing, and timing of initiation. While maternal supplementation has been associated with improved pregnancy outcomes [1,4,11,14], less is known about its direct effects on maternal and infant micronutrient status. Examining changes in nutritional status is important to better understand the biological pathways through which supplementation exerts its effects and to determine whether improvements in pregnancy outcomes are mediated by enhanced micronutrient status. This insight can inform the development of more effective, targeted interventions. This review synthesized evidence on vitamin supplementation (single or combined) and, where relevant, multiple micronutrient supplements or lipid-based nutrient supplements’ (LNS) impact on maternal (serum and milk) and infant (serum) nutritional status in LMICs, exploring factors like supplement dosage, duration and maternal subgroups influencing effectiveness.

## Methods

### Eligibility criteria

Our synthesis included randomized controlled trials (RCTs) involving healthy (i.e., non-diseased) pregnant and/or lactating women of any age and parity, assigned either individually or in clusters to receive a vitamin-containing intervention (e.g., single, double, or multiple vitamins, MMS, or LNS), without restrictions on year, sample size or intervention frequency. Eligible studies assessed these interventions in various forms (e.g., capsules, drops, syrup, or powder) and compared dosages or formulations. While this review focuses on vitamin supplementation, we included studies of MMS and LNS because these formulations contain vitamins and are widely used in LMIC settings. This approach allowed us to assess whether vitamins delivered as part of multi-component supplements achieve similar effects as standalone vitamin interventions. However, we note that the inclusion of MMS/LNS limits our ability to isolate the effects of individual vitamins due to potential interactions with minerals or fatty acids. To mitigate this, we conducted subgroup analyses where possible (e.g., comparing MMS to placebo or IFA) and interpreted the findings with caution, emphasizing outcomes directly related to vitamin status.

Control groups included iron, iron and folic acid, placebo, usual care, or no supplementation. Outcomes measured maternal and/or infant micronutrient status or milk concentrations via biochemical tests. Only English-language articles were included.

We excluded non-randomized studies (e.g., quasi-experiments, uncontrolled before-after studies), observational studies, editorials, and those focusing on special populations (e.g., women with anemia, HIV, tuberculosis, or metabolic disorders). Studies on fortified foods, or those solely assessing pregnancy outcomes (e.g., low birth weight, preterm birth, small-for-gestational-age birth, perinatal death, stillbirth, neonatal death, or maternal and infant mineral status) without biochemical measures of maternal or infant nutritional status, were also excluded.

### Data sources and search strategy

We searched MEDLINE (PubMed), EMBASE, CENTRAL (Cochrane Library), and the WHO library database from the inception through December 23, 2024, using keywords, indexing, and free-text terms. Additional searches were conducted on ClinicalTrials.gov, UNICEF, WHO, and World Bank websites. Detailed PubMed search terms are in Online Supplementary Table S1. References from key articles and reviews were manually checked. Records were managed using EndNote X9 (Clarivate Analytics, Pennsylvania, United States) and Covidence (Veritas Health Innovation, Melbourne, Australia).

### Study selection

Two reviewers independently screened titles/abstracts for eligibility, eliminating irrelevant studies. Full texts of potentially eligible studies were assessed independently by at least two reviewers, with disagreements resolved through discussion. The selection process followed the Preferred Reporting Items for Systematic Reviews and Meta-Analyses (PRISMA) guidelines, documenting reasons for exclusions [15].

### Data extraction

Two reviewers independently extracted data using a pilot-tested form, resolving disagreements through discussion. Extraction details included study characteristics (title, authors, year, country and setting, funding, design), participant demographics (sample size, age, pregnancy status), intervention specifics (timing, duration, dose, vitamin content), comparator/control, sample collection timing, outcomes, and findings (point estimates and variance measures). For continuous outcomes, we extracted baseline and/or post-intervention mean concentrations, standard deviations, and measurement units where available. These data were used to assess comparability across studies and to determine whether standardized or mean differences were appropriate for meta-analysis.

### Risk of bias assessment

Two reviewers independently assessed the risk of bias using Cochrane Risk-of-Bias Version 2 [16], resolving disagreements through discussion. For RCTs, five domains were evaluated for individually randomized trials, with an extra domain for cluster trials [17].

### Data synthesis

All included studies were summarized in text and table format, categorized into water- and fat-soluble vitamins. Random-effects, inverse variance-weighted meta-analyses were conducted for outcomes reported in ≥2 studies with consistent intervention, comparator, and outcome definitions. The random-effects method accommodated heterogeneity in micronutrient supplements regarding dose, duration, and target populations. The generic inverse-variance approach adjusted study weights for both continuous and binary outcomes based on the effect estimate variance. Vitamin doses were standardized to International Units (IUs). Meta-analyses focused on the vitamin status of maternal, infant and cord serum as well as milk vitamin composition. Subgroup analyses explored maternal status, supplement type, duration, and dosage. For studies reporting timing of supplementation, we extracted available data on specific trimester of initiation during pregnancy (first [<14 weeks], second [14-28 weeks], or third [>28 weeks]), and postpartum initiation timing (days/weeks after delivery). Where sufficient data were available (≥2 studies with consistent timing reporting), we conducted subgroup analyses to examine whether initiation timing or duration modified treatment effects. Timing data were synthesized descriptively when meta-analysis was not feasible due to inconsistent reporting across studies. Other potential subgroup analyses (e.g., co-interventions, region, and age), were limited by insufficient data.

For continuous outcomes, we used standardized mean differences (SMDs) with 95% confidence intervals (CIs) when studies assessed the same outcome using different measurement scales or units (e.g., serum vitamin A reported in μmol/L vs. μg/dL). When outcomes were reported using the same units across studies, we used mean differences (MDs) to preserve interpretability. All outcomes were oriented so that positive effect sizes consistently indicated improvements in micronutrient status (e.g., higher serum concentrations), and negative effect sizes indicated deterioration. When necessary, we multiplied mean values or effect estimates by –1 to align directionality across studies before pooling. Binary outcomes were represented as odds ratios with 95% CIs.

For studies with insufficient data, we contacted corresponding authors for additional statistics. If unavailable, studies were retained but excluded from meta-analysis. Heterogeneity was assessed using Higgins' I^2^, with 50-90% indicating substantial and >90% considerable variability. Funnel plots evaluated publication bias for outcomes with ≥5studies. Meta-analyses were conducted with STATA Software Version 17, with significance set at p<0.05.

### Assessment of certainty of evidence

The overall certainty of evidence was assessed using the Grading of Recommendation, Assessment, Development and Evaluation (GRADE) approach, evaluating risk of bias, publication bias, imprecision, inconsistency, and directness [18]. Evidence was categorized as high, moderate, low, or very low certainty.

### Registration and reporting

The study protocol is available elsewhere [19] and registered on PROSPERO (ID: CRD42022308715). Reporting followed the PRISMA guidelines [15].

## Results

### Basic characteristics of the included studies

**Figure 1:**
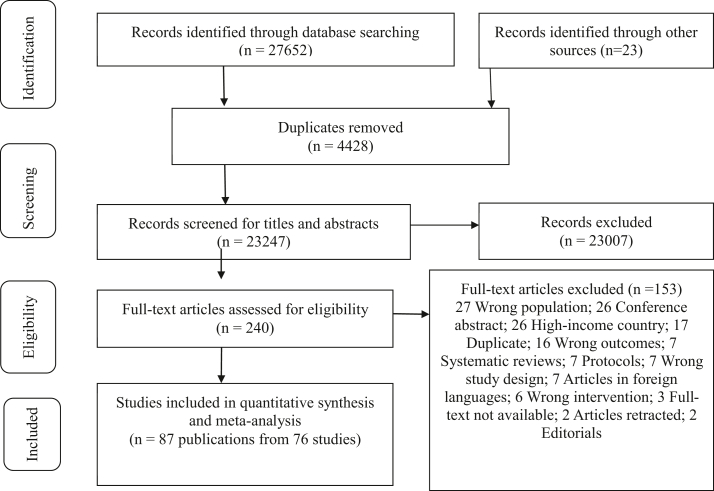
**Flow chart for the literature search for intervention**.

From 27,675 records identified, 27,435 were excluded after deduplication and title/abstract screening. Of 240 full-text articles reviewed, 153 were excluded based on various criteria, leaving 87 for analysis (Figure 1). A narrative synthesis was conducted for all included articles, with characteristics summarized in Online Supplementary Table S2 and briefly described below. Meta-analyses on the impact of micronutrients on maternal and infant nutritional status and their effects on micronutrient concentrations in milk and cord serum are conducted when feasible and summarized in Table 1.

**Table 1:** Summary of meta-analyses findings by vitamins– overall and sub-groups

Outcome	Subgroup of interventions	Standardized Mean Difference or Odds Ratio (95% CI)	*P* value
**Water-soluble vitamins**			
**Thiamin (B-1) studies**			
Vitamin B-1 level in milk	All studies supplementing B-1	0.31 (-0.11, 0.73)	0.15
	Vitamin B-1 2.4 mg – 2.8 mg dose	0.26 (-0.02, 0.53)	0.07
**Riboflavin (B-2) studies**			
Vitamin B-2 level in milk	All studies supplementing B-2	0.64 (-0.11, 1.39)	0.09
**Folate (B-9) studies**			
Vitamin B-9 concentration in cord serum	All studies supplementing folate	0.08 (-0.07, 0.23)	0.29
**Cobalamin (B-12) studies**			
Vitamin B-12 concentration in maternal serum	All studies supplementing vitamin B12	0.39 (0.11, 0.68)	0.01
	Excluding supplementation from preconception	0.41 (0.06, 0.76)	0.020
	Vitamin B-12 only	0.66 (0.06, 1.26)	0.03
	MMS containing vitamin B-12	0.23 (0.11, 0.36)	<0.001
	2 – 3 ug of vitamin B-12	0.28 (0.10, 0.46)	<0.001
	Vitamin B-12 concentration categorized as =/<150 pmol/L	0.43 (0.19, 0.95)	0.040
Vitamin B-12 level in milk	All studies supplementing vitamin B12	0.33 (0.02, 0.63)	0.040
	Vitamin B-12 only	0.65 (-0.24, 1.54)	0.150
	MMS and/or LNS containing vitamin B-12	-0.08 (-0.35, 0.18)	0.530
	Vitamin B-12 in pregnancy only	0.14 (-0.14, 0.42)	0.320
	50 ug vitamin B-12	-0.02 (-0.39, 0.36)	0.93
	Vitamin B-12 concentration categorized as =/<310 pmol/L	0.66 (0.45, 0.96)	0.030
Vitamin B-12 concentration in infant serum	All studies supplementing vitamin B12	0.80 (0.28, 1.32)	<0.001
	Vitamin B-12 concentration categorized as =/<150 pmol/L	0.56 (0.24, 1.34)	0.19
Vitamin B-12 concentration in cord serum	All studies supplementing vitamin B-12	0.45 (-0.12, 1.01)	0.12
	Vitamin B-12 concentration categorized as =/<150 pmol/L	0.53 (0.36, 0.78)	<0.001
**Fat-soluble vitamins**			
**Vitamin A studies**			
Vitamin A concentration in maternal serum	All studies supplementing vitamin A	0.60 (0.13, 1.08)	<0.001
	Vitamin A only	0.28 (-0.02, 0.58)	0.07
	Retinol or retinyl palmitate only	0.32 (-0.08, 0.72)	0.12
	Retinol only	0.37 (-0.56, 1.29)	0.44
	Retinyl palmitate only	0.20 (-0.23, 0.62)	0.36
	Beta carotene only	0.19 (0.07, 0.32)	<0.001
	Vitamin A supplementation only in pregnancy	0.95 (0.05, 1.86)	0.04
	Vitamin A supplementation only in postpartum	0.17 (0.02, 0.32)	0.03
	Single dose of Vitamin A (200,000 IU – 400,000 IU)	0.13 (-0.03, 0.29)	0.11
	Continuous dose of vitamin A	0.83 (0.17, 1.49)	0.01
	Vitamin A supplementation compared to placebo	0.43 (0.21, 0.65)	<0.001
	Vitamin A concentration categorized as =/<1.05umol/L	0.61 (0.51, 0.73)	<0.001
	Vitamin A concentration categorized as =/<0.7umol/L	0.55 (0.43, 0.71)	<0.001
Vitamin A level in milk	All studies supplementing vitamin A	0.82 (-0.09, 1.73)	0.08
	Vitamin A only	0.53 (0.19, 0.86)	<0.001
	Retinyl palmitate only	0.18 (-0.04, 0.40)	0.10
	Vitamin A supplementation only in pregnancy	0.05 (-0.33, 0.43)	0.78
	Vitamin A supplementation only in postpartum	1.14 (-0.12, 2.40)	0.08
	Single dose of Vitamin A (200,000 IU – 400,000 IU)	0.53 (0.23, 0.82)	<0.001
	Vitamin A supplementation compared to placebo	0.59 (0.33, 0.85)	<0.001
	Vitamin A concentration categorized as =/<1.05umol/L	1.02 (0.36, 2.87)	0.97
	Vitamin A concentration categorized as =/<0.7umol/L	0.50 (0.11, 2.30)	0.37
Vitamin A concentration in infant serum	All studies supplementing vitamin A	0.55 (-0.07, 1.17)	0.08
	Vitamin A only	0.39 (-0.05, 0.82)	0.08
	Retinol or retinyl palmitate only	0.17 (-0.10, 0.44)	0.21
	Retinyl palmitate only	0.11 (-0.18, 0.40)	0.44
	Vitamin A supplementation only in pregnancy	2.16 (-0.45, 4.78)	0.10
	Vitamin A supplementation only in postpartum	0.26 (-0.06, o.58)	0.11
	Single dose of Vitamin A (200,000 IU – 400,000 IU)	0.68 (-0.15, 1.51)	0.11
	Vitamin A supplementation compared to placebo	0.33 (-0.03, 0.69)	0.07
	Vitamin A deficiency =/<0.7umol/L	0.74 (0.37, 1.48)	0.40
Vitamin A concentration in cord serum	All studies supplementing vitamin A	0.10 (-0.09, 0.29)	0.29
**Vitamin D studies**			
Vitamin D concentration in maternal serum	All studies supplementing vitamin D	1.52 (0.98, 2.07)	<0.001
	Vitamin D only	1.91 (1.19, 2.63)	<0.001
	Vitamin D supplementation in pregnancy only	1.68 (0.99, 2.37)	<0.001
	Vitamin D supplementation in postpartum only	0.69 (0.35, 1.04)	<0.001
	Daily dose of =/< 2, 000 IU vitamin D	0.98 (0.47, 1.49)	<0.001
	Daily dose of >2,000 IU vitamin D	2.83 (1.91, 3.75)	<0.001
	Vitamin D supplementation compared to placebo	1.92 (1.10, 2.74)	<0.001
	Vitamin D concentration categorized as =/<50nmol/L	0.30 (0.14, 0.64)	<0.001
Vitamin D concentration in infant serum	All studies supplementing vitamin D	1.29 (0.32, 2.25)	0.01
	Vitamin D supplementation in pregnancy only	0.23 (-0.09, 0.55)	0.11
	Vitamin D supplementation in postpartum only	2.28 (0.75, 3.81)	<0.001
	Vitamin D supplementation compared to placebo	1.35 (-0.03, 2.73)	0.06
	Vitamin D concentration categorized as =/<50nmol/L	0.20 (0.06, 0.72)	0.01
Vitamin D concentration in cord serum	All studies supplementing vitamin D	2.09 (0.93, 3.25)	<0.001
	Vitamin D only	2.50 (1.30, 3.70)	<0.001
	Vitamin D supplementation compared to placebo	3.22 (2.54, 3.90)	<0.001
	Vitamin D concentration categorized as =<50nmol/L	0.25 (0.03, 2.37)	0.23
**Vitamin E studies**			
Alpha tocopherol concentration in maternal serum	All studies supplementing vitamin E	0.54 (-0.48, 1.56)	0.30
	Vitamin E supplementation in pregnancy only	0.14 (0.05, 0.23)	<0.001
	Vitamin E supplementation in postpartum only	1.37 (-1.20, 3.95)	0.30
Gamma Tocopherol concentration in maternal serum	All studies supplementing vitamin E	-0.20 (-0.80, 0.40)	0.51
Alpha tocopherol level in milk	All studies supplementing Vitamin E	2.05 (1.73, 2.38)	<0.001
	Vitamin E only	2.17 (1.77, 2.57)	<0.001

CI: Confidence Intervals; IFA: Iron and Folic Acid Supplementation; IU: International Units; LNS: Lipid-Based Nutrient Supplements; MMS: Multiple Micronutrient Supplements

The 87 articles represent 76 independent trials [[20], [21], [22], [23], [24], [25], [26], [27], [28], [29], [30], [31], [32], [33], [34], [35], [36], [37], [38], [39], [40], [41], [42], [43], [44], [45], [46], [47], [48], [49], [50], [51], [52], [53], [54], [55], [56], [57], [58], [59], [60], [61], [62], [63], [64], [65], [66], [67], [68], [69], [70], [71], [72], [73], [74], [75], [76], [77], [78], [79], [80], [81], [82], [83], [84], [85], [86], [87], [88], [89], [90], [91], [92], [93], [94], [95], [96], [97], [98], [99], [100], [101], [102], [103], [104], [105], [106]], including three clustered-RCTs [61, 90, 102] and 73 individual RCTs. Studies were conducted in 20 countries, with the highest numbers in India (n=12), Brazil (n=11), Bangladesh (n=10), and Iran (n=9). Two multi-country studies included Ghana, India, and Peru, and one study spanned Ghana and Malawi (Table S2).

Among 76 studies, 12 administered water-soluble vitamins (6 B vitamins, 5 IFA, and 1 vitamin C), 51 provided fat-soluble vitamins (20 vitamin A, 26 vitamin D, 4 vitamin E, and 1 vitamins A and D), nine provided MMS, and four provided lipid-based micronutrient supplements (Table S2).

Among the 76 studies, 45 supplemented pregnant women, 27 lactating women, and four both lactating women and infant dyads. of these, 27 assessed only maternal nutritional status, eight focused solely on infant nutritional status, 11 examined effects on milk, and one evaluated cord serum. Additionally, 12 studies assessed both maternal and infant nutritional status, seven evaluated maternal nutritional status and effects on milk, three examined infant nutritional status and effects on milk, and seven assessed maternal and infant nutritional status as well as effects on milk (Table S2).

### Main results

#### Water-soluble vitamins

##### The B vitamins

Six studies provided B vitamins: three daily vitamin B-12 [45,46,88]; and one each daily vitamins B-1 [50], B-2 [27], or B-complex [61]. Studies of vitamins B-1 and B-2 involved pregnant women; the remaining four involved lactating women. Vitamin B-12 dosages ranged from 2 μg to 250 μg/day. Five studies used placebo controls; one had no intervention as a control group (Table S2).

Five studies, covered by six articles, examined folic acid supplementation in pregnant women, one beginning in the first trimester [28] and four in the second [35, 42, 47, 48, 105]. The daily folic acid doses varied from 0.35 mg to 5 mg, often with iron [35, 47, 48, 105], or iron and/or multiple micronutrients [28, 42]. One study compared IFA supplementation with placebo [28], while others compared it with either an iron-rich diet [47], vitamin A alone [35], or MMS including IFA [42, 48, 105] (Table S2).

Although three studies evaluated the effects of folic acid supplementation on maternal folate concentrations, a meta-analysis was not conducted because only one study [35] met the inclusion criteria for assessing changes in serum folate concentrations between supplemented and true control groups. Of the remaining two studies, one was excluded due to unclear group definitions [47], while the other lacked a valid control arm as all intervention groups received comparable amounts of folate [105]. The eligible study reported a significant increase of approximately 25 nmol/L in serum folate concentrations among participants who received folic acid alone (400 μg daily), folic acid (60 mg) with iron, or a multiple micronutrient supplement, compared with those who received only vitamin A. Additionally, maternal folic acid supplementation during pregnancy did not reduce the risk of folate deficiency, defined as serum levels ≤ 6.8 nmol/L (Table 1). A single study examined the impact of daily supplementation with 400 μg of folic acid and 30 or 60 mg of iron from 14 weeks of gestation to 3 months postpartum, on infant serum folate concentrations, finding no significant differences between supplementation groups [48]. Similarly, maternal folic acid supplementation, either alone or with other nutrients, had no effect on folate concentrations in cord serum (Table 1). None of the included studies assessed the impact of folic acid supplementation on folate levels in milk.

**Figure 2:**
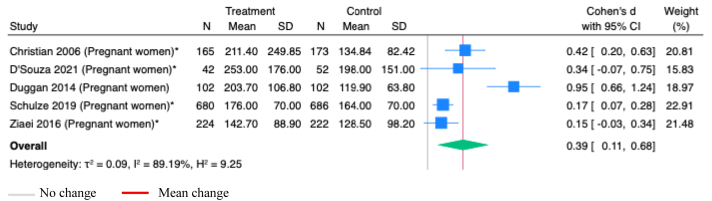
**Random-effects meta-analysis of supplementation of B-12 alone or with other micronutrients during pregnancy on B-12 concentration in maternal serum (pmol/L)**. ∗Studies administered MMS; others administered vitamins only

Vitamin B-12 supplementation during pregnancy, alone or with other micronutrients, significantly increased maternal serum cobalamin concentrations (SMD 0.39; 95% CI 0.11, 0.68; *P*=0.01; Studies=5; Figure 2). However, the estimates showed substantial heterogeneity (I^2^=89.19%), and the asymmetry in funnel plot suggested potential publication bias (Figure S1).

Maternal supplementation of vitamin B-12 during pregnancy, alone or as a part of MMS, significantly reduced the risk of maternal cobalamin deficiency defined as ≤ 150 pmol/L (OR 0.43; 95%CI 0.19, 0.95; *P*=0.040; Studies=6) (Table 1). Maternal vitamin B-12 supplementation during pregnancy increased infant serum cobalamin concentrations in two studies (Figure S2). However, no significant reduction in infant vitamin B-12 deficiency (≤ 150 pmol/L) was observed (OR 0.56; 95% CI 0.24, 1.34; *P*=0.19; Studies=2; Table 1). Maternal supplementation of B-12 during pregnancy had no significant impact on cobalamin concentrations in cord serum (Figure S3). However, a significant reduction in cord serum vitamin B-12 deficiency (≤ 150 pmol/L) was noted (OR 0.53; 95% CI 0.36, 0.78; *P*=<0.001; Studies=4; Figure S4).

Vitamin B-12 supplementation during pregnancy or extending into the postpartum period significantly increased cobalamin concentrations in milk (SMD 0.33; 95% CI 0.02, 0.063; *P*=0.04; I^2^=79.35%; Studies=5; Figure S5). However, a study from India included in the meta-analysis found significantly higher cobalamin concentrations in milk at 6 weeks postpartum (at the end of supplementation), but not at 3 or 6 months postpartum [46]. Additionally, vitamin B-12 supplementation as part of LNS or MMS during pregnancy alone or extending into the postpartum period showed no significant impact on cobalamin concentrations in milk (SMD -0.08; 95% CI -0.35, 0.18; *P*=0.53; Studies=3; I^2^=27.10%; Figure S6). Similarly, vitamin B-12 supplementation solely during pregnancy (SMD 0.14; 95% CI -0.14, 0.42; *P*=0.32; Studies=3; Figure S7) and supplementation at a dose of 50 μg extending into the postpartum period (SMD -0.02; 95% CI -0.39, 0.36; *P*=0.93; Studies=2; I^2^=66.02%; Figure S8) showed no significant effects. However, vitamin B-12 supplementation alone showed a non-significant trend toward increased milk cobalamin concentrations (SMD 0.65; 95% CI -0.24, 1.54; *P*=0.15; Table 1). Four studies assessing cobalamin concentrations in milk, using a deficiency cut-off of ≤ 310 pmol/L, showed that supplementation was associated with lower odds of deficiency (OR 0.66; 95% CI 0.45, 0.96; *P*=0.03; Figure S9).

**Figure 3:**
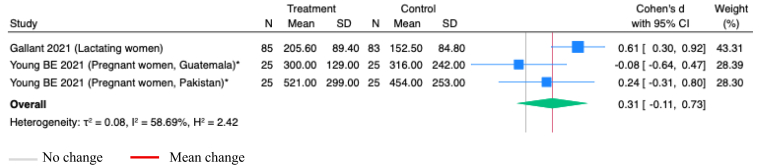
**Random-effects meta-analysis of vitamin B-1 supplementation during pregnancy or postpartum on vitamin B-1 concentrations in milk, comparing with the group receiving no supplementation (Ug/L)**. ∗Studies administered LNS; others administered vitamins only

Studies of vitamin B-1 supplementation did not evaluate its effect on thiamine concentrations in maternal or infant serum. Supplementation of vitamin B-1, either alone or combined with other micronutrients during pregnancy, with samples assessed 2 weeks postpartum, or postpartum, with samples assessed at 2, 4, 12 and 24 weeks, had no significant impact on thiamine concentrations in milk (SMD 0.31; 95% CI -0.11, 0.73; *P*=0.15; Studies=2; I^2^=58.69%; Figure 3).

**Figure 4:**
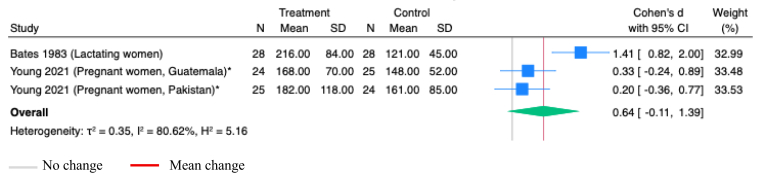
**Random-effects meta-analysis of vitamin B-2 supplementation during pregnancy or postpartum on vitamin B-2 concentrations in milk, comparing with the group receiving no supplementation (Ug/L)**. ∗Studies administered LNS; others administered vitamins only

Studies on vitamin B-2 supplementation did not assess its effects on riboflavin concentrations in maternal or infant serum. Supplementation with vitamin B-2 alone in lactating women, with sample collected on day 84 of supplementation [27], or in combination with other vitamins during pregnancy, with samples collected 2 weeks postpartum [105], show no significant impact on riboflavin concentrations in milk (SMD 0.64; 95% CI -0.11, 1.39; *P*=0.09; Studies=2; I^2^=80.62%; Figure 4).

##### Vitamin C

One study assessed the effects of 2,000 IU daily vitamin C supplementation versus placebo during pregnancy. Between weeks 20 and 36, the mean leukocyte vitamin C concentration decreased from 17.5 to 15.23 μg/10^8^ cells in the placebo group but increased from 17.26 to 22.17 μg/10^8^ cells in the supplemented group, with significant within- and between-group differences [33]. None of the included studies examined the effects of vitamin C supplementation during pregnancy or lactation on infant serum or milk vitamin C concentrations.

#### Fat-soluble vitamins

##### Vitamin A

Among the 20 vitamin A studies, 13 provided it exclusively to lactating women (within 48 hours to 6 weeks after delivery, targeting vitamin A repletion in early lactation) [20,26,29, 30,44,52,65,78,83,91,94,95,97,100,106], four to pregnant women (mostly in the second or third trimester) [69,70,85,93,102,103], and three to both pregnant women and their infants [23,24,64]. The doses varied widely, reflecting different supplementation strategies including WHO-recommended high-dose bolus supplementation for postpartum women and alternative daily or weekly regimens for pregnant women. Doses included: a single 200,000 IU dose, two 200,000 IU doses 24 hours apart, a single dose of 200,000 IU before and 10 days after delivery, a single 400,000 IU dose within 24 hours of delivery, weekly doses of 4,800 IU retinol equivalents with or without IFA, weekly doses of 7,000 μg retinol equivalents, daily doses of 8,000 IU vitamin A as retinyl palmitate, and a daily dose of 0.5 mg (approximately 1667 IU) of vitamin A. Comparison groups included a placebo, no intervention, or the national IFA supplementation program. (Table S2).

**Figure 5:**
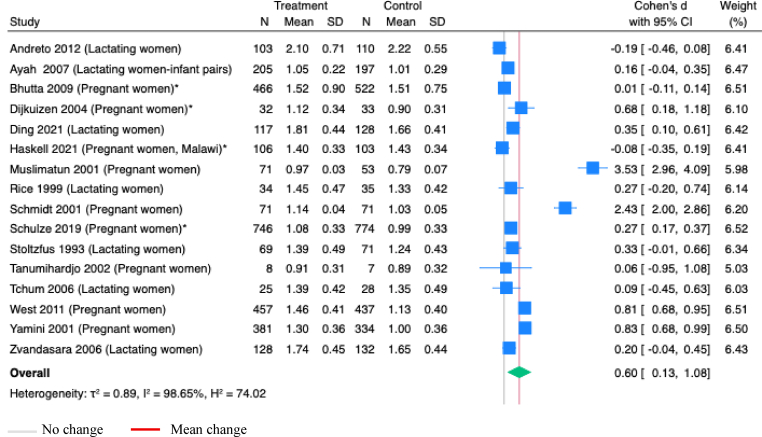
**Random-effects meta-analysis of supplementation of vitamin A during pregnancy or postpartum on retinol or retinyl palmitate concentration in maternal serum (μmol/L)**. ∗Studies administered multiple micronutrients; others administered vitamins only

Maternal serum vitamin A concentrations were assessed in 18 studies, with 17 included in meta-analyses due to consistent outcome definitions. Vitamin A supplementation during pregnancy or postpartum, alone or with other micronutrients, significantly increased retinol or retinyl palmitate concentration in maternal serum by 0.60 μmol/L (95% CI 0.13, 1.08; *P*<0.001; Studies=16; Figure 5). However, considerable heterogeneity was observed (I^2^=98.65%). An asymmetrical funnel plot (Figure S10) suggested potential publication bias.

**Figure 6:**
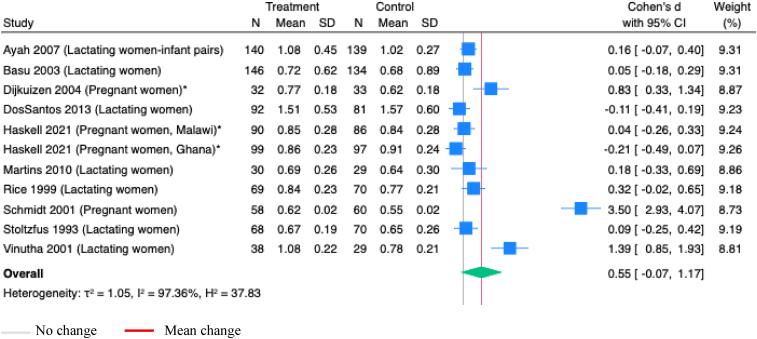
**Random-effects meta-analysis of maternal supplementation of vitamin A during pregnancy or postpartum on infant serum vitamin A (μmol/L)**. ∗Studies administered multiple micronutrients; others administered vitamins only

Retinol or retinyl palmitate supplementation during pregnancy or postpartum, vitamin A supplementation alone during pregnancy or postpartum, or single dose of vitamin A supplementation during postpartum showed no impact on maternal serum vitamin A concentrations (Figure S11-S13). However, beta-carotene supplementation alone during pregnancy or postpartum, vitamin A supplementation during pregnancy, vitamin A supplementation during postpartum, a continuous dose of vitamin A supplementation during pregnancy or postpartum, or vitamin A supplementation during pregnancy or postpartum compared to placebo only showed significant effects on maternal serum vitamin A concentrations (Table 1 & Supplementary Figures S14-S18). Vitamin A supplementation during pregnancy or postpartum, alone or with other nutrients, reduced vitamin A deficiency at ≤ 0.7 μmol/L (OR 0.55; 95% CI 0.43, 0.71; *P*<0.001; Studies=5; Figure S19) and at ≤ 1.05 μmol/L levels (OR 0.61; 95% CI 0.51, 0.73; *P*<0.001; Studies=7; Figure S20). Maternal vitamin A supplementation during pregnancy or postpartum did not significantly increase infant serum vitamin A concentrations (SMD 0.55; 95% CI -0.07, 1.17; *P*=0.08; Studies=10; I^2^=97.36%; Figure 6).

Maternal supplementation with retinol and retinyl palmitate during pregnancy or postpartum, or only vitamin A supplementation during postpartum did not improve vitamin A concentrations in infant serum (Figures S21 & S22). This lack of improvement was consistent across interventions with retinol, beta-carotene, or retinyl palmitate alone, as well as vitamin A supplementation solely during pregnancy (Table 1). Additionally, a single dose of vitamin A supplementation did not improve infant serum vitamin A concentrations, nor did it reduce infant serum vitamin A deficiency below the <0.7 μmol/L threshold (Table 1 & Figure S23).

**Figure 7:**
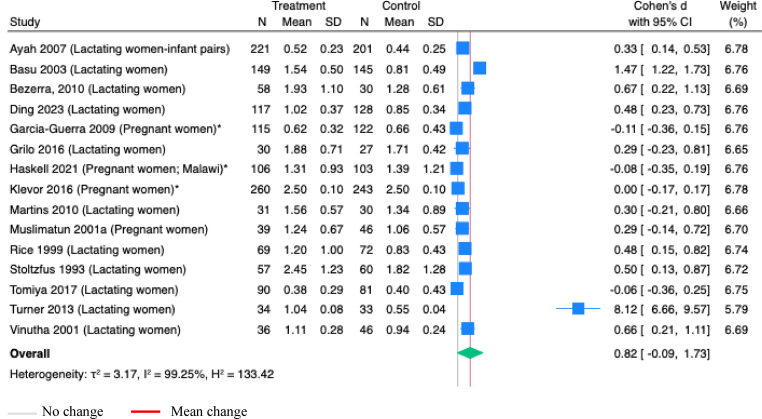
**Random-effects meta-analysis of maternal supplementation of vitamin A alone or with other nutrients during pregnancy or postpartum on vitamin A concentrations in milk (μmol/L)**. ∗Studies administered MMS; others administered vitamins only

Vitamin A concentrations in milk were assessed in 20 studies, with 15 having consistent outcome definitions for meta-analyses. Maternal vitamin A supplementation during pregnancy or postpartum, alone or combined with other nutrients, did not significantly increase vitamin A milk concentrations (SMD 0.82; 95%CI: -0.09, 1.73; *P*=0.08; I^2^=99.25%; Figure 7).

**Figure 8:**
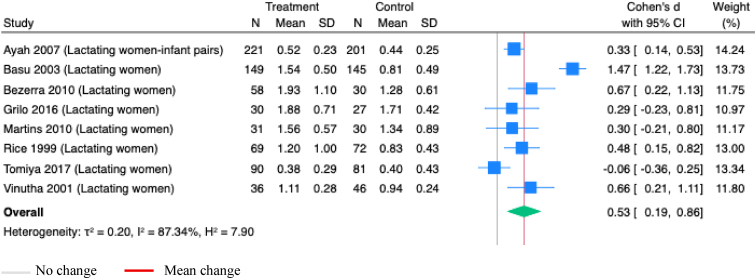
**Random-effects meta-analysis of maternal supplementation of vitamin A alone during postpartum on the vitamin A concentrations in milk (μmol/L)**. Note: All studies administered vitamins only

Maternal supplementation with vitamin A alone or combined with other nutrients to only lactating women did not significantly increase vitamin A concentrations in milk (SMD 1.14; 95%CI: -0.12, 2.40; *P*=0.08; Studies=11; I^2^=99.34%; Figure S24). However, vitamin A supplementation alone during the postpartum period significantly increased vitamin A concentrations in milk (SMD 0.53; 95%CI: 0.19, 0.86; *P*<0.001; Studies=8; I^2^=87.34; Figure 8). Similarly, a single dose of vitamin A (200,000 IU to 400,000 IU) given to lactating women significantly improved vitamin A concentrations in milk (SMD 0.53; 95%CI: 0.23, 0.82; *P*<0.001; Studies=9; I^2^= 85.52%; Figure S25). Supplementation with vitamin A to lactating women, compared to placebo, also showed a significant increase in the vitamin A concentrations in milk (SMD 0.59; 95% CI 0.33, 0.85; *P* <0.001; Studies=9; I^2^= 81.75%; Figure S26), but no improvements were observed in vitamin A levels in milk (Figures S27 to S28), measured at ≤0.7 μmol/L and ≤1.05 μmol/L (Table 1). Maternal vitamin A supplementation during pregnancy had no effect on vitamin A concentrations in cord serum (Table 1).

##### Vitamin D

Twenty-five studies, represented by 31 articles, examined the effects of vitamin D supplementation. Of these, 17 studies administered vitamin D during pregnancy [21,22,25,37, 39,41,49,53,54,56,58,[66], [67], [68],^71^,^73^,^77^,^79^,^80^,^84^,^89^,^98^,^99^], seven studies during postpartum [32, 34,72,74,76,82,96], and one study during both pregnancy and postpartum [81]. Among the pregnancy studies, most began in the second trimester (10 studies), followed by the third trimester (4 studies) and first trimester (3 studies). Dosages varied widely: daily doses ranged from 200 IU to 4,000 IU, weekly doses from 400 IU and 35,000 IU, and monthly doses from 12,000 IU to 120,000 IU. Two studies tested fortnightly doses of 50,000 IU. Postpartum interventions typically began within 1 week to 3 months after delivery, with high-dose regimens such as 60,000 IU over 10 days. Eleven studies compared vitamin D supplementation with placebos, 10 compared different dosing regimens, two focused on infant supplementation without maternal supplementation, and one used sun exposure or routine care as the control group (Table S2).

**Figure 9:**
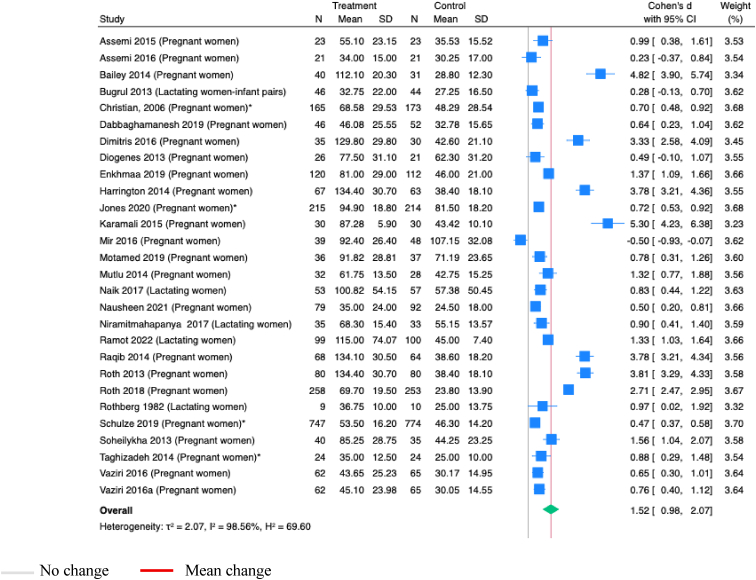
**Random-effects meta-analysis of maternal vitamin D supplementation during pregnancy or postpartum on the vitamin D concentrations in maternal serum (nmol/L)**. ∗Studies administered MMS; others administered vitamins only

Thirty-two studies assessed maternal serum vitamin D concentrations, with 21 using consistent outcome definitions, allowing meta-analysis. Supplementation of vitamin D during pregnancy or postpartum, alone or with other micronutrients, significantly increased maternal serum vitamin D concentrations (SMD 1.52; 95% CI 0.98, 2.07; *P*<0.001; Figure 9), with considerable heterogeneity in the estimates (I^2^=98.56%), and an asymmetrical funnel plot (Figure S29), indicating possible publication bias.

**Figure 10:**
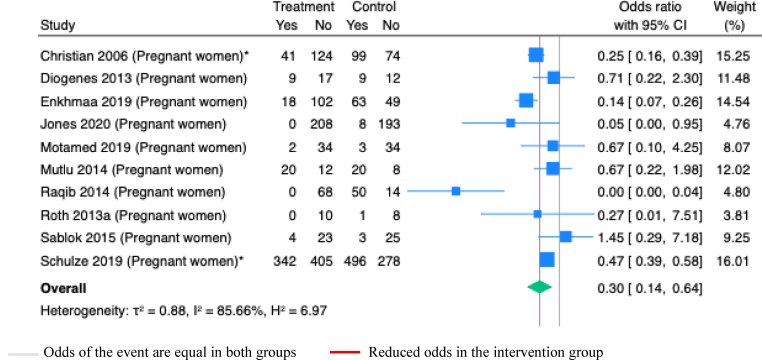
**Random-effects meta-analysis of maternal vitamin D supplementation during pregnancy on vitamin D deficiency in maternal serum (<50nmol/L). “Yes” indicates deficiency**. ∗Studies administered MMS; others administered vitamins only

Maternal supplementation with vitamin D alone during pregnancy or postpartum, in combination with other micronutrients at various doses during pregnancy or lactation, during only pregnancy, or during only postpartum significantly improved maternal serum vitamin D concentrations (Figures S30 – S34). Supplementation of vitamin D alone or with other micronutrients, compared to placebo, also resulted in a statistically significant increase in maternal serum vitamin D concentrations (Table 1). Furthermore, vitamin D supplementation during pregnancy significantly reduced maternal vitamin D deficiency at ≤50 nmol/L level (OR 0.30; 95% CI 0.14, 0.64; *P*<0.001; Studies=10; I^2^=85.66%; Figure 10). None of the included studies assessed the effects of maternal vitamin D supplementation on milk vitamin D concentrations.

**Figure 11:**
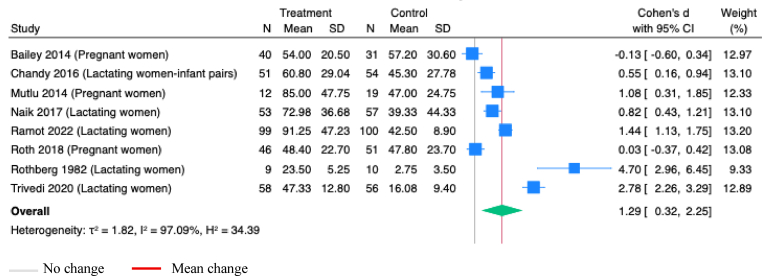
**Random-effects meta-analysis of maternal vitamin D supplementation during pregnancy or postpartum on the vitamin D concentration in infant serum (nmol/L)**. Note: All studies administered vitamins only

Maternal vitamin D supplementation, alone or with other micronutrients, during pregnancy or postpartum, showed a statistically significant increase in infant serum vitamin D concentrations (SMD 1.29; 95% CI 0.32, 2.25; *P*=0.01; Studies=8; I^2^=97.09%; Figure 11). Maternal vitamin D supplementation solely during postpartum also demonstrated significant improvements in infant serum vitamin D concentrations (Table 1). In a meta-analysis involving four studies of vitamin D supplementation during pregnancy or postpartum, either alone or combined with other micronutrients, infants with vitamin D deficiency (≤ 50 nmol/L) were significantly likely to be in the control group than in the intervention group (OR 0.20; 95% CI 0.06, 0.72; *P*=0.01; I^2^=78.55%; Figure S35).

Maternal vitamin D supplementation, alone or with other micronutrients, significantly increased cord serum vitamin D concentrations (SMD 2.09; 95% CI 0.93, 3.25; *P*<0.001; Studies=8; I^2^=98.47%; Figure S36). However, no significant difference was observed in vitamin D deficiency (≤ 50 nmol/L) in cord serum between the intervention and control groups (OR 0.25; 95% CI 0.03, 2.37; *P*=0.23; Studies=5; I^2^=96.85%; Figure S37).

##### Vitamins E

Four studies, all in Brazil, investigated vitamin E supplementation compared to no intervention [36,38,62,75]. Among these, three studies provided a single dose of 400,000 IU RRR-α-tocopherol, while one administered a single dose of 800,000 IU RRR-α-tocopherol to lactating women. Additionally, a single study provided daily oral doses of 1,800 IU vitamin A and 600 IU vitamin D-2 for 2 months to lactating women, comparing it with placebo (Table S2) [40].

Two outcomes, maternal serum alpha tocopherol and breastmilk alpha tocopherol, were analyzed in two or more independent studies with consistent outcome definitions, enabling meta-analysis. In the meta-analysis of five studies, vitamin E supplementation resulted in a non-significant increase of 0.54 μmol/L (95% CI -0.48, 1.56; *P*=0.30; I^2^=99.21%; Table 1) in maternal serum alpha tocopherol concentrations and a significant average increase of 2.05 μmol/L (95% CI 1.73, 2.38; *P*<0.001; Table 1) in milk tocopherol concentrations.

##### Multiple micronutrient supplementation (MMS)

Eight studies, represented by eleven articles, administered daily MMS [31,35,51,55,57, ,60,63,86,87,92,101]. These studies used diverse formulations: two provided MMS with 800 μg retinol equivalent and 17 micronutrients, one utilized the UNIMMAP formulation, one followed UNICEF recommendations, one exceeded 100-150% of the recommended dietary allowance (RDA) for multiple micronutrients, one included a multivitamin with various vitamins, one provided multivitamins containing 50 μg of vitamin B-12, and another provided 10 multivitamins. Six studies compared MMS with daily IFA supplementation, one with a placebo, one with or without iron, and one with MMS containing minerals. Five studies started MMS supplementation during pregnancy (in the first or second trimester), three continued supplementations from pregnancy into postpartum, and one provided MMS exclusively during postpartum (Table S2).

##### Lipid-based nutrient supplement (LNS)

Five studies examined the impact of LNS [43,55,60,90,104]. Four of these studies provided LNS to pregnant women until six months postpartum, while one study provided it only during pregnancy. The composition and dosages of vitamins in the LNS varied across studies. Four studies provided continuous LNS supplementation, whereas one study provided a single 30-gm dose with multiple vitamins or divided the 30-gm dose into three doses. Two studies compared LNS with no intervention groups, two with IFA supplements, and one with a routine program including monthly growth monitoring and nutrition education (Table S2). Due to considerable heterogeneity in supplementation protocols and measured outcomes across studies, a meta-analysis was not performed, as combining results was not feasible.

### Risk of Bias and Certainty of Evidence

Among the 84 articles based on 73 individual RCTs, 55 were judged as having a high risk of bias, 17 to have some concerns, and one to have a low risk of bias (Table S3). The primary sources of bias for individual RCTs included missing outcome data (25 articles with a high risk), issues with randomization and selection of the reported results (14 articles each with a high risk), measurement bias (8 articles with a high risk), and deviations from the intended intervention (2 articles with a high risk). For the three articles based on cluster RCTs, two were judged to have a high risk of bias, and one had some concerns. According to the GRADE approach (Table S4), this meta-analysis provides low-certainty evidence due to study bias, publication bias, and heterogeneity.

## Discussion

This systematic review and meta-analysis synthesized the impact of single or combined vitamin supplementation or multiple micronutrient supplementation during pregnancy and/or lactation on maternal and infant nutritional status in LMICs. Our results indicate that vitamin A supplementation, alone or combined with other micronutrients during pregnancy and postpartum, may improve vitamin A concentrations in maternal serum and milk. Similarly, vitamin B-12 supplementation, alone or as part of MMS, could increase vitamin B-12 concentrations in maternal and infant serum and milk. Vitamin D supplementation may increase vitamin D concentrations in maternal, infant and cord serum. This is the first systematic review evaluating effects of single, double, or multiple micronutrient supplementation on these outcomes in LMICs based on effectiveness studies.

Our study found that vitamin A supplementation during pregnancy and lactation increased maternal serum vitamin A concentrations and reduced deficiency, with a stronger effect during pregnancy. A single high dose of vitamin A improved vitamin A concentrations in milk but did not increase maternal serum vitamin A concentrations, likely due to preferential partitioning of vitamin A from maternal circulation to breast milk rather than accumulation in maternal serum. For infants, the lack of effect on serum vitamin A concentrations is likely explained by the temporal mismatch between intervention timing and outcome assessment, i.e., bolus doses were administered in the early postpartum period (first 1-2 weeks), but infant serum vitamin A status was typically assessed at 3-6 months postpartum, which may have missed the peak serum response following supplementation.. However, maternal vitamin A supplementation during pregnancy or postpartum, either alone or with other micronutrients, did not increase infant or cord serum vitamin A concentrations. Our results are consistent with prior systematic reviews and meta-analyses demonstrating modest increases in maternal serum retinol [4,[107], [108], [109], [110], [111]].

While the WHO previously recommended vitamin A supplementation in deficient populations, current WHO antenatal care guidelines (2016) [112] focus primarily on pregnancy interventions. Our findings of modest increases in maternal serum retinol during pregnancy align with evidence supporting targeted vitamin A interventions in deficient populations during pregnancy [113, 114], though the evidence base for postpartum supplementation remains limited. Timing appears critical, with earlier and sustained interventions likely yielding better maternal status. Further high-quality research is needed to clarify the role of vitamin A supplementation in improving maternal and infant outcomes.

B vitamins are essential for fetal development, supporting energy metabolism, neural function, and overall well-being [115]. Pyridoxine aids neural development and neurotransmitter synthesis, while folic acid is essential for preventing neural tube defects. Cobalamin deficiency is associated with adverse maternal and neonatal outcomes [116]. However, the impact of B vitamins supplementation during pregnancy on maternal and infant nutritional status remains uncertain. Our study found no significant increase in thiamine concentrations in milk with supplementation alongside other micronutrients, though a Cambodian RCT showed increased milk thiamine concentrations with extended postpartum vitamin B-1 supplementation [50]. Similarly, our study observed a slight but statistically insignificant increase in thiamine and riboflavin concentrations in milk with vitamins B-1 and B-2 supplementation.

For folic acid, a meta-analysis of maternal serum folate concentrations was not conducted because only one study met the eligibility criteria, while our study found marginal, non-significant, effects on cord serum folate concentrations when supplemented alongside other micronutrients during pregnancy and lactation. However, a cluster-RCT in Nepal found that pregnant women receiving folic acid alone (400 μg), folic acid with iron (60 mg), or MMS (with 400 μg folic acid and 60 mg iron) had significantly higher serum folate concentrations (∼25 nmol/L) compared to those receiving vitamin A alone [35]. Additionally, a small trial in Mexico among postpartum women reported improved folate concentrations with daily MMS (400 μg folic acid) with or without iron [59].

For vitamin B-12, our study found increased cobalamin concentrations in maternal, infant, and cord serum, as well as in milk, with supplementation during pregnancy and postpartum. A recent systematic review of five trials suggests that vitamin B-12 supplementation during pregnancy may reduce maternal vitamin B-12 deficiency risk, with uncertain evidence from two trials and three trials noted increased maternal vitamin B-12 concentrations in the third trimester or at three months postpartum, compared to placebo or no supplementation [116]. Our review, noting the absence of comparable systematic reviews on vitamin B-12 supplementation during pregnancy and its impact on maternal and infant B-12 status, emphasizes the importance of administering vitamin B-12 in at-risk groups, such as vegans, vegetarians, and food-insecure populations. Clinically, higher maternal and infant vitamin B12 concentrations may aid neural development and lower neurodevelopmental delays risks, particularly where animal-source food intake is low. Further research is needed to clarify the benefits of B vitamin supplementation.

Our systematic review highlights the positive effects of vitamin D supplementation during pregnancy and postpartum on maternal, infant, and cord serum concentrations of 25-hydroxyvitamin D [25(OH)D]. Maternal supplementation, alone or with other micronutrients, significantly increased maternal 25(OH)D concentrations compared to controls, with higher doses (>2000 IU/day) showing greater benefits, particularly in women with baseline levels ≤ 50 nmol/L. Infant and cord serum in intervention groups also had significantly increased 25(OH)D concentrations. Postpartum vitamin D supplementation resulted in higher infant serum 25(OH)D concentrations. These findings align with previous reviews [4,[118], [119], [120], [121]] and important for clinical benefits in reducing rickets and supporting bone and immune health. However, study heterogeneity, small sample sizes, and baseline 25(OH)D variations warrant cautious interpretation. More trials are needed to establish pregnancy-specific thresholds and guide policy.

Combining multiple micronutrients into a single supplement is proposed as a cost-effective strategy to improve maternal health during pregnancy [122]. Evidence from large meta-analyses of randomized trials and individual patient data from LMICs shows that MMS reduces the risk of low birth weight, small for gestational age births, preterm birth, and neonatal mortality, especially among undernourished and anemic women, while lipid based nutrient supplements have shown limited benefits [123,124]. However, our review found limited evidence that supplementation strategies (including single nutrients, combinations, or MMS formulations) consistently reduced micronutrient deficiencies, with mixed effects observed across different vitamins. Specifically, while some MMS studies in our analysis showed improvements in individual vitamin biomarkers (e.g., vitamin B-12, vitamin D), the pooled analyses that included both MMS and single nutrient interventions revealed inconsistent effects on deficiency reduction for vitamins A and folate. The largest MMS trial in Bangladesh showed that while daily MMS reduced adverse birth outcomes and decreased the prevalence of deficiencies by 15%–40% compared with IFA at 32-week gestation, it did not eliminate existing deficiencies, nor did it further reduce anemia prevalence or prevent more women from becoming deficient by the third trimester, suggesting higher need in later gestation [86]. These findings underscore the importance of early initiation (e.g., preconception or first trimester) and adequate dosing to address deficiencies, especially in high-prevalence settings. Further research is needed to understand the effect of the current formulation of MMS in addressing micronutrient deficiencies during pregnancy.

The reported beneficial effects of MMS in reducing the risks of small-for-gestational-age and low birth weight [7,123,124,125], support the shift from maternal IFA to MMS with IFA in antenatal programs. The biomarkers examined in our review may offer critical insights into optimal dosing strategies, suggesting that higher doses in multiple RDA contributed to improved status compared to single RDA. For example, vitamin D doses >2,000 IU/day were more effective than ≤2,000 IU/day in improving maternal serum concentrations, while vitamin B-12 supplementation at 50-250 μg was effective for improving maternal status but higher doses may be needed to consistently impact infant outcomes. Determining the optimal composition, bioavailability, and dosing of micronutrients in MMS remains a research priority [127,128]. However, this optimization must navigate a critical tension: balancing adequate dosing against safety concerns.

On one hand, while the UNIMMAP formulation is widely used and deemed safe when paired with an adequate diet, concerns have been raised about exceeding tolerable upper intake levels for certain micronutrients, specifically iron, niacin and folate, which can reach 127%, 103% and 100% of the upper levels, respectively, when MMS is combined with other interventions [129]. On the other hand, a single RDA may be insufficient to correct deficiencies in malnourished populations, particularly in South Asia and sub-Saharan Africa. In Nepal, vitamin B-12 deficiency persisted despite RDA-level supplementation from pregnancy through six months postpartum [35]. Similarly, HIV-positive women showed nutrient inadequacies even with supplements exceeding the RDA for B vitamins [131]. In Guinea-Bissau, doubling the RDA of UNIMMAP improved birth outcomes, though biomarker data were lacking [132]. To address such gaps, bioefficacy studies in Bangladesh and Kenya are evaluating optimal micronutrient doses for women of reproductive age and pregnant women. These include dose-response trials using pharmacokinetic modeling to identify intake levels that ensure sufficiency without excess [133,134].

Nutrient-nutrient interactions may partly explain the variable biomarker responses seen with MMS compared to single or dual micronutrient interventions. While MMS targets multiple deficiencies, certain nutrients can interfere with each other’s absorption or metabolism. For example, high iron can reduce zinc absorption, and excess folate may mask vitamin B-12 deficiency. Fat-soluble vitamins (e.g., A, D, E) and B-complex vitamins may also compete for uptake or share metabolic pathways, potentially blunting individual nutrient effects [130]. These interactions may underlie the mixed biomarker outcomes for nutrients like folate, thiamine, and vitamin A in MMS. Future research should explore nutrient bioavailability and metabolic interactions to optimize MMS formulations.

The role of micronutrient supplementation and the optimal dose for women during lactation also warrants further investigation. Evidence from postnatal maternal MMS trials is limited, with a Cochrane review identifying only two small RCTs among HIV-negative women, neither assessed maternal or infant micronutrient status [135]. In Tanzania, multivitamin supplementation containing 50μg of vitamin B-12 from mid-pregnancy through six weeks postpartum showed a modest, non-significant increase in breast milk B-12 levels at six weeks postpartum, with 70% of women still having inadequate levels despite supplementation, suggesting that higher doses or alternative supplementation strategies may be needed in deficiency-prone settings [63]. In contrast, trials in Ghana [60] and Iran [92], found no significant effects of MMS or multivitamins on breast milk vitamin A or maternal and infant vitamin D status, respectively.

The findings from this review have important implications for maternal nutrition policy and antenatal supplementation programs in LMICs. While evidence from large trials and meta-analyses indicates that MMS can improve birth outcomes and maternal nutritional status compared to IFA supplementation alone, the evidence on its broader impact on micronutrient deficiencies and long-term maternal and infant health remains limited and variable. Therefore, policy decisions to transition to MMS in antenatal programs should consider local nutritional needs, health system capacity, and cost-benefit implications. Policymakers are encouraged to adopt a phased or context-specific approach, integrating MMS within strengthened antenatal care frameworks, while prioritizing ongoing monitoring and evaluation to generate further evidence on effectiveness, and program feasibility. Complementary strategies, such as dietary diversification and food fortification, should also be emphasized to address persistent deficiencies. Implementation research is essential to optimize MMS formulation, dosing, timing, and delivery models tailored to diverse populations and settings.

Our review has several limitations. First, diversity in interventions, timing, outcomes, measures, and assessments may lead to difficulty in meta-analyzing the results effectively. Second, subgroup analyses were limited by inconsistent data availability and could not be conducted based on factors like age, parity, baseline nutritional status of mothers, supplementation timing (e.g., trimester of initiation or postpartum duration), intervention duration, and timing of outcome assessment. In addition, composition of interventions could not be compared for some subgroup analyses (for example, UNIMMAP vs IFA vs MMS formulations). Third, most of the trials included in our study were not designed to assess the impact of vitamin or multiple micronutrient supplementation on the maternal and infant vitamin status and these outcomes were measured as secondary outcomes. Due to this fact, there is a higher chance of getting a random positive or false negative result by chance. Additionally, given the small number of MMS studies, pooling them with single nutrient interventions limited our ability to draw definitive conclusions about the specific effectiveness of MMS formulations compared to single nutrient approaches, though we conducted subgroup analyses where data permitted. Finally, some studies conducted subgroup analyses within the main trials. While informative, these analyses, particularly when focused on secondary outcomes, can increase the risk of false positives, especially if subgroups are no longer balanced and the benefits of randomization are compromised. Moreover, the pooling of studies with varying doses, durations, and co-formulated nutrients introduces interpretive challenges. While meta-analysis provides an overall estimate of effect, it may obscure meaningful differences in individual trial contexts, potentially overstating the uniformity of benefits.

## Conclusions

Vitamin supplementation is crucial for improving maternal and infant nutrition in LMICs, though our findings showed its varied effects. Water-soluble vitamins like B-12 improved maternal serum concentrations and reduced deficiency rates, its impact on infant B-12 levels, cord blood and milk concentrations was inconsistent. Folic acid had minimal effects on maternal and infant folate status and milk folate concentrations. Among fat-soluble vitamins, vitamin A improved maternal serum and milk concentrations postpartum, while vitamin D significantly enhanced both maternal and infant serum concentrations during pregnancy or postpartum. These findings suggest that supplementation strategies can improve biochemical markers of nutritional status, but their translation into meaningful clinical outcomes may depend on contextual factors such as underlying deficiency prevalence, baseline dietary intake, and health service delivery capacity.

Research on MMS have demonstrated greater effectiveness compared to IFA alone, aligning evidence linking MMS to better birth outcomes, including reduced low birth weight and small-for-gestational-age risks, and improved maternal nutritional. However, outcome variability highlights further research. The WHO recommendation highlights the need for both clinical and implementation research. Clinical studies might explore optimal dosing of iron and B vitamins in MMS formulation for addressing micronutrient deficiencies in antenatal care. Evidence from this review suggests single RDA levels may be inadequate for B vitamins in populations with high baseline deficiency prevalence, while concerns remain about iron potentially exceeding tolerable upper intake levels when combined with other vitamins. Implementation research should assess factors like feasibility, acceptability, equity, cost-effectiveness, and program delivery. For many countries, the most practical step is to conduct implementation research to strengthen antenatal care and evaluate MMS rollout, including its potential to improve antenatal care attendance.

## Author contributions

The authors’ responsibilities were as follows – SS, DW and WF: conceptualized and designed the research; SS and CAY: designed the study protocol, searches and assembled the search results; SS, CAY, MY, and LN: conducted full-text screening, data extraction and risk of bias assessment. SS and CAY: performed statistical analysis. SS: wrote the paper. All authors read and critically edited the paper. All authors approved the final paper.

## Data sharing

Data described in the manuscript, extraction sheets and analytical codes will be made available upon reasonable request.

## Author contributions

The authors’ responsibilities were as follows – SS, DW and WF: conceptualized and designed the research; SS and CAY: designed the study protocol, searches and assembled the search results; SS, CAY, MY, and LN: conducted full-text screening, data extraction and risk of bias assessment. SS and CAY: performed statistical analysis. SS: wrote the paper. All authors read and critically edited the paper. All authors approved the final paper.

## Declaration of Generative AI and AI-assisted technologies in the writing process

The authors confirm that no generative AI or AI-assisted technologies were used in performing data analysis, generating scientific content, writing, editing, or preparation of this manuscript.

All content was conceived, developed, and written solely by the authors.

## Funding

This work is financially supported by a grant from the Bill and Melinda Gates Foundation (INV-037672). The funder had no role in developing the protocol, designing and conducting the study or reporting the findings.

## Conflict of interest

The authors have no competing interests to declare.
