# Supplementary material for: Effects of vitamin and multiple micronutrient supplementation for pregnant and/or lactating women on maternal and infant nutritional status in low- and middle-income countries: a systematic review and meta-analysis
Source: Adv Nutr. 2025 Aug 5;16(12):100487. doi: 10.1016/j.advnut.2025.100487 (PMC12766057; doi:10.1016/j.advnut.2025.100487)
Supplement: Multimedia component 1 [file mmc1.docx]

**Supplementary Figure 1: Random-effects meta-analysis of maternal supplementation of vitamin B-12 alone or in combination with other micronutrients on cobalamin content in maternal serum (pmol/L).**

Funnel plot of standard error by difference in mean

**Supplementary Figure 2: Random-effects meta-analysis of maternal supplementation of vitamin B-12 during pregnancy on vitamin B-12 content in infant serum (pmol/L)**

Note: All studies administered vitamins only

No change Mean change

**Supplementary Figure 3: Random-effects meta-analysis of maternal supplementation of vitamin B-12 during pregnancy on cobalamin content in cord serum (pmol/L).**

*Studies administered MMS; others administered vitamins only

No change Mean change

**Supplementary Figure 4: Random-effects meta-analysis of maternal supplementation of vitamin B-12 on cobalamin deficiency in cord serum (≤ 150 pmol/L). “Yes” indicates cobalamin status ≤ 150 pmol/L.**

*Studies administered MMS; others administered vitamins only

Odds of the event are equal in both groups Reduced odds in the intervention group

**Supplementary Figure 5: Random-effects meta-analysis of maternal supplementation of vitamin B-12 alone or in combination with other micronutrients during pregnancy or extending from pregnancy to postpartum on cobalamin content in milk (pmol/L).**

*Studies administered multiple vitamins; **studies administered LNS; others administered vitamins only

No change Mean change

**Supplementary Figure 6: Random-effects meta-analysis of maternal supplementation of vitamin B-12 as part of LNS or MMS on cobalamin content in milk (pmol/L).**

*Studies administered multiple vitamins; **studies administered LNS

No change Mean change

**Supplementary Figure 7: Random-effects meta-analysis of maternal supplementation of vitamin B-12 during pregnancy on cobalamin content in milk (pmol/L).**

*Studies administered LNS; others administered vitamins only

No change Mean change

**Supplementary Figure 8: Random-effects meta-analysis of maternal supplementation of vitamin B-12 in a dose of 50 ug on cobalamin content in milk (pmol/L).**

*Studies administered multiple vitamins

No change Mean change

**Supplementary Figure 9: Random-effects meta-analysis of vitamin B-12 supplementation only or with other vitamin on vitamin B-12 levels in milk (≤ 310 pmol/L) comparing with placebo or no supplementation. “Yes” indicates a vitamin B-12 status ≤ 310pmol/L.**

*Studies administered multiple vitamins; **studies administered LNS

Odds of the event are equal in both groups Reduced odds in the intervention group

**Supplementary Figure 10: Random-effects meta-analysis of supplementation of vitamin A during pregnancy or postpartum on retinol or retinyl palmitate concentration in maternal serum (µmol/L)**.

Funnel plot of standard error by difference in mean

**Supplementary Figure 11: Random-effects meta-analysis of supplementation of retinol or retinyl palmitate supplementation during pregnancy or postpartum on retinol or retinyl palmitate concentration in maternal serum (µmol/L).**

*Studies administered MMS; others administered vitamins only

No change Mean change

**Supplementary Figure 12: Random-effects meta-analysis of supplementation of vitamin A supplementation alone during pregnancy or postpartum on vitamin A concentration in maternal serum (µmol/L).**

*Studies administered MMS; others administered vitamins only

No change Mean change

**Supplementary Figure 13: Random-effects meta-analysis of supplementation of single dose vitamin A supplementation during postpartum on vitamin A concentration in maternal serum (µmol/L).**

Note: All studies administered vitamins only

No change Mean change

**Supplementary Figure 14: Random-effects meta-analysis of supplementation of beta-carotene only during pregnancy or postpartum on beta-carotene concentration in maternal serum (µmol/L).**

Note: All studies administered vitamins only

No change Mean change

**Supplementary Figure 15: Random-effects meta-analysis of supplementation of vitamin A alone or in combination with other nutrients during pregnancy on vitamin A concentration in maternal serum (µmol/L).**

*Studies administered MMS; others administered vitamins only

No change Mean change

**Supplementary Figure 16: Random-effects meta-analysis of supplementation of vitamin A alone or in combination with other nutrients during postpartum on vitamin A concentration in maternal serum (µmol/L).**

Note: All studies administered vitamins only

No change Mean change

**Supplementary Figure 17: Random-effects meta-analysis of daily supplementation of vitamin A during pregnancy or postpartum on vitamin A concentration in maternal serum (µmol/L).**

*Studies administered MMS; others administered vitamins only

No change Mean change

**Supplementary Figure 18: Random-effects meta-analysis of daily supplementation of vitamin A during pregnancy or postpartum, in comparison with placebo, on vitamin A concentration in maternal serum (µmol/L).**

Note: All studies administered vitamins only

No change Mean change

**Supplementary Figure 19: Random-effects meta-analysis of supplementation of vitamin A on maternal serum vitamin A deficiency <0.7 µmol/L; “Yes” indicates deficiency.**

*Studies administered MMS; others administered vitamins only

Odds of the event are equal in both groups Reduced odds in the intervention group

**Supplementary Figure 20: Random-effects meta-analysis of supplementation of vitamin A on maternal serum vitamin A categorized at <1.05 µmol/L level; “Yes” indicates deficiency.**

*Studies administered MMS; others administered vitamins only

Odds of the event are equal in both groups Reduced odds in the intervention group

**Supplementary Figure 21: Random-effects meta-analysis of maternal supplementation of retinol or retinyl palmitate during pregnancy or postpartum on vitamin A concentration in infant serum (µmol/L).**

Note: All studies administered vitamins only

No change Mean change

**Supplementary Figure 22: Random-effects meta-analysis of maternal supplementation of vitamin A supplementation alone during postpartum on vitamin A concentration in infant serum (µmol/L).**

Note: All studies administered vitamins only

No change Mean change

**Supplementary Figure 23: Random-effects meta-analysis of maternal supplementation of vitamin A during pregnancy or postpartum on infant serum vitamin A deficiency ≤ 0.7 µmol/L; “Yes” indicates deficiency.**

*Studies administered MMS; others administered vitamins only

Odds of the event are equal in both groups Reduced odds in the intervention group

**Supplementary Figure 24: Random-effects meta-analysis of maternal supplementation of vitamin A alone or with other nutrients during postpartum on vitamin A content in milk (µmol/L).**

Note: All studies administered vitamins only

No change Mean change

**Supplementary Figure 25: Random-effects meta-analysis of maternal supplementation of single dose vitamin A (in the range of 200,000 IU to 400,000 IU) during postpartum on the vitamin A content in milk (µmol/L).**

Note: All studies administered vitamins only

No change Mean change

**Supplementary Figure 26: Random-effects meta-analysis of maternal supplementation of vitamin A during postpartum and compared with placebo on the vitamin A content in milk (µmol/L).**

Note: All studies administered vitamins only

No change Mean change

**Supplementary Figure 27: Random-effects meta-analysis of maternal supplementation of vitamin A during pregnancy or postpartum on vitamin A levels in milk at <0.7 µmol/L.**

*Studies administered MMS; others administered vitamins only

Odds of the event are equal in both groups Reduced odds in the intervention group

**Supplementary Figure 28: Random-effects meta-analysis of maternal supplementation of vitamin A on vitamin A levels at <1.05 µmol/L.**

*Studies administered MMS; others administered vitamins only

Odds of the event are equal in both groups No difference in the odds between the intervention and control groups

**Supplementary Figure 29: Random-effects meta-analysis of maternal supplementation of vitamin D alone or in combination with other micronutrients on vitamin D concentration in maternal serum (nmol/L).**

Funnel plot of standard error by difference in mean

**Supplementary Figure 30: Random-effects meta-analysis of maternal supplementation of vitamin D only during pregnancy or postpartum on vitamin D concentration in maternal serum (nmol/L).**

Note: All studies administered vitamins only; No change Mean change

**Supplementary Figure 31: Random-effects meta-analysis of maternal supplementation of vitamin D (less than 2000IU/day) during pregnancy or lactation on vitamin D concentration in maternal serum (nmol/L).**

*Studies administered MMS; others administered vitamins only; No change Mean change

**Supplementary Figure 32: Random-effects meta-analysis of maternal supplementation of vitamin D (more than 2000IU/day) to pregnant and lactating women on vitamin D concentration in maternal serum (nmol/L).**

Note: All studies administered vitamins only

No change Mean change

**Supplementary Figure 33: Random-effects meta-analysis of maternal supplementation of vitamin D during pregnancy on vitamin D concentration in maternal serum (nmol/L).**

*Studies administered MMS; others administered vitamins only; No change Mean change

**Supplementary Figure 34: Random-effects meta-analysis of maternal supplementation of vitamin D during postpartum on vitamin D concentration in maternal serum (nmol/L).**

Note: All studies administered vitamins only

No change Mean change

**Supplementary Figure 35: Random-effects meta-analysis of maternal supplementation of vitamin D on vitamin D deficiency in infant serum (≤ 50nmol/L). “Yes” indicates deficiency.**

Note: All studies administered vitamins only

Odds of the event are equal in both groups Reduced odds in the intervention group

**Supplementary Figure 36: Random-effects meta-analysis of maternal supplementation of vitamin D alone or in combination with other on vitamin D content in cord serum (nmol/L).**

*Studies administered MMS; others administered vitamins only

No change Mean change

**Supplementary Figure 37: Random-effects meta-analysis of maternal supplementation of vitamin D alone or in combination with other nutrients on vitamin D concentration in cord serum (≤ 50 nmol/L).**

*Studies administered MMS; others administered vitamins only

Odds of the event are equal in both groups Reduced odds in the intervention group

**Online Supplementary Material**

**Supplementary Table S1: Detailed search terms used for the systematic review**

| **No.** | **Concept** | **Search terms** |
| --- | --- | --- |
| #1 | Trials | ‘Clinical Trials as Topic’[Mesh Terms] OR ‘Randomized Controlled Trial’[pt] OR Clinical Trial[pt] OR ‘Controlled Clinical Trial’[pt] OR ‘randomized controlled trials as topic’[MeSH terms] OR ‘controlled trial’ OR intervention OR ‘random allocation’[MeSH terms] OR random OR trial OR ‘Clinical Study’[pt] OR ‘Clinical Studies as Topic’[Mesh terms] OR ‘Therapeutic Uses’[Mesh Terms] OR ‘therapeutic use’[Subheading] |
| #2 | Intervention | ‘micronutrients’ [MeSH Terms] OR ‘micronutrients’ [All Fields] OR ‘micronutriments’ [All Fields] ‘micronutrient’ [All Fields] OR (‘dietary’ [All Fields] AND ‘supplements’ [All Fields]) OR ‘dietary supplements’ [All Fields] OR ‘supplement’ [All Fields] OR ‘supplements’ [All Fields] OR ‘supplemented’ [All Fields] OR ‘supplementing’ [All Fields] OR ‘supplement s’ [All Fields] OR ‘vitamins’ [MeSH Terms] OR ‘vitamins’ [All Fields] OR ‘vitamin’ [All Fields] OR ‘multi-vitamin’ [All Fields] OR ‘multivitamin’ [All Fields] OR ‘multivitamins’ [All Fields] OR ‘vitamin a’ [MeSH Terms] OR ‘vitamin a’ [All Fields] OR ‘retinol’ [All Fields] OR ‘retinols’ [All Fields] OR ‘vitamin b complex’ [MeSH Terms] OR ‘vitamin b complex’ [All Fields] OR ‘vitamin b’ [All Fields] OR ‘B-complex’ [All Fields] OR ‘thiamine’ [MeSH Terms] OR ‘thiamine’ [All Fields] OR ‘thiamin’ [All Fields] OR ‘thiamines’ [All Fields] OR (‘vitamin’ [All Fields] AND ‘b1’ [All Fields]) OR ‘vitamin b1’ [All Fields] OR ‘riboflavin’ [MeSH Terms] OR ‘riboflavin’ [All Fields] OR (‘vitamin’ [All Fields] AND ‘b2’ [All Fields]) OR ‘vitamin b2’ [All Fields] OR ‘niacinamide’ [MeSH Terms] OR ‘niacinamide’ [All Fields] OR ‘niacin’ [Mesh Terms] OR ‘niacin’ [All Fields] OR (‘vitamin’ [All Fields] AND ‘b3’ [All Fields]) OR ‘vitamin b3’ [All Fields] OR ‘pantothenic acid’ [MeSH Terms] OR (‘pantothenic’ [All Fields] AND ‘acid’ [All Fields]) OR ‘pantothenic acid’ [All Fields] OR (‘vitamin’ [All Fields] AND ‘b5’ [All Fields]) OR ‘vitamin b5’ [All Fields] OR ‘pyridoxine’ [MeSH Terms] OR ‘pyridoxine’ [All Fields] OR ‘pyridoxine’ [All Fields] OR ‘pyridoxines’ [All Fields] OR (‘vitamin’ [All Fields] AND ‘b6’ [All Fields]) OR ‘vitamin b6’ [All Fields] OR ‘biotin’ [MeSH Terms] OR ‘biotin’ [All Fields] OR (‘vitamin’ [All Fields] AND ‘b7’ [All Fields]) OR ‘vitamin b7’ [All Fields] OR ‘folic acid’ [MeSH Terms] OR (‘folic’ [All Fields] AND ‘acid’ [All Fields]) OR ‘folic acid’ [All Fields] OR ‘folate’ [All Fields] OR ‘folates’ [All Fields] OR (‘vitamin’ [All Fields] AND ‘b9’ [All Fields]) OR ‘vitamin b9’ [All Fields] OR ‘vitamin b 12’ [MeSH Terms] OR ‘vitamin b 12’ [All Fields] OR ‘cyanocobalamin’ [All Fields] OR ‘cyanocobalamine’ [All Fields] OR ‘ascorbic acid’ [MeSH Terms] OR (‘ascorbic’ [All Fields] AND ‘acid’ [All Fields]) OR ‘ascorbic acid’ [All Fields] OR ‘vitamin c’ [All Fields] OR ‘cholecalciferol’ [MeSH Terms] OR ‘cholecalciferol’ [All Fields] OR ‘cholecalciferols’ [All Fields] OR ‘vitamin d’ [MeSH Terms] OR ‘vitamin d’ [All Fields] OR ‘calcifediol’ [MeSH Terms] OR ‘calcifediol’ [All Fields] OR ‘25-hydroxyvitamin D’ [All Fields] OR ‘ 25 hydroxyvitamin d’ [All Fields] OR ‘ergocalciferols’ [MeSH Terms] or ‘ergocalciferols’ [All fields] OR ‘vitamin e’ [MeSH Terms] OR ‘vitamin e’ [All Fields] OR ‘vitamin k 1’ [MeSH Terms] OR ‘vitamin k 1’ [All Fields] OR ‘phytomenadione’ [All Fields] |
| #3 | Pregnant women and reproductive age women | ‘gravidity’[MeSH Terms] OR ‘gravidity’[All Fields] OR ‘pregnant’ [All Fields] OR ‘pregnants’ [All Fields] OR ‘pregnant women’ [MeSH Terms] OR (‘pregnant’ [All Fields] AND ‘women’ [All Fields]) OR ‘pregnant women’ [All Fields]) AND ‘pregnancy’ [MeSH Terms]) OR ‘pregnancy’ [All Fields] OR ‘gestation’ [All Fields] OR ‘gestational’ [All Fields] OR (‘gestate’ [All Fields] OR ‘gestated’ [All Fields] OR ‘gestates’ [All Fields] OR ‘gestating’ [All Fields] OR ‘gestational’ [All Fields] OR ‘gestations’ [All Fields] OR ‘pregnancy’ [MeSH Terms] OR ‘pregnancy’ [All Fields] OR ‘gestation’ [All Fields]) OR ‘mothers’ [MeSH Terms] OR ‘mothers’ [All Fields] OR ‘mother’ [All Fields] OR ‘mothering’ [All Fields] OR ‘mothered’ [All Fields] OR ‘maternal’ [All Fields] OR ‘maternally’ [All Fields] OR ‘maternity’ [All Fields] OR ‘maternities’ [All Fields] OR ‘maternal age’ [MeSH Terms] OR (‘maternal’ [All Fields] AND ‘age’ [All Fields]) OR ‘maternal age’ [All Fields] OR ‘child bearers’ [All Fields] OR ‘childbearing’ [All Fields] OR ‘prenatal care’ [MeSH Terms] OR (‘prenatal’ [All Fields] AND ‘care’ [All Fields]) OR ‘prenatal care’ [All Fields] OR ‘perinatal care’ [MeSH Terms] OR (‘perinatal’ [All Fields] AND ‘care’ [All Fields]) OR ‘perinatal care’ [All Fields] OR ‘anti-natal’ [All Fields] OR ‘antenatal’ [All Fields] OR ‘pre-partum’ [All Fields] OR (‘pre’ [All Fields] AND ‘partum’ [All Fields]) OR ‘perinatology’ [MeSH Terms] OR ‘perinatology’ [All Fields] OR ‘peri-partum’ [All Fields] OR (‘peri’ [All Fields] AND ‘partum’ [All Fields]) OR ‘lactating’ [All Fields] OR ‘lactation’ [MeSH Terms] OR ‘lactation’ [All Fields] OR ‘breast feeding’ [MeSH Terms] OR (‘breast’ [All Fields] AND ‘feeding’ [All Fields]) OR ‘breast feeding’ [All Fields] OR ‘lactational’ [All Fields] OR ‘lactations’ [All Fields] OR ‘lactators’ [All Fields]) AND (‘women’ [All Fields] OR ‘women’ [MeSH Terms] OR ‘women’ [All Fields] OR ‘woman’ [All Fields] OR (‘reproductive’ [All Fields] AND ‘age’ [All Fields] AND ‘women’ [MeSH Terms] OR ‘women’ [All Fields] OR ‘woman’ [All Fields]) |
| #4 | Low- and middle-income countries | ‘Developing Countries’[MeSH Terms] OR ‘developing country’ OR ‘developing nation’ OR ‘less developed country’ OR ‘less developed nation’ OR ‘third world nation’ OR ‘third world country’ OR ‘under developed nation’ OR ‘underdeveloped nation’ OR ‘under developed country’ OR ‘underdeveloped country’ OR ‘middle income country’ OR ‘middle-income country’ OR ‘middle income nation’ OR ‘middle-income nation’ OR ‘low income country’ OR ‘low-income country’ OR ‘low income nation’ OR ‘low-income nation’ OR ‘poor country’ OR ‘poor nation’ OR LMIC OR lmics OR ‘Africa’[MeSH] OR ‘Asia’[MeSH] OR ‘South America’[MeSH Terms] OR ‘Latin America’[MeSH terms] OR ‘Central America’[MeSH Terms] OR ‘Africa’ OR ‘Asia’ OR ‘South America’ OR ‘Latin America’ OR ‘Central America’ OR ‘Afghanistan’ OR ‘Albania’ OR ‘Algeria’ OR ‘Samoa’ OR ‘Angola’ OR ‘Armenia’ OR ‘Azerbaijan’ OR ‘Bangladesh’ OR ‘Bengali’ OR ‘Belarus’ OR ‘Belize’ OR ‘Benin’ OR ‘Bhutan’ OR ‘Bolivia’ OR ‘Bosnia’ OR ‘Herzegovina’ OR ‘Botswana’ OR ‘Brazil’ OR ‘Bulgaria’ OR ‘Burkina Faso’ OR ‘Burkinabe’ OR ‘Burundi’ OR ‘Cabo Verd’ OR ‘Cape Verd’ OR ‘Cambodia’ OR ‘Cameroon’ OR ‘Central African’ OR Chad OR China OR Chinese OR Colombia OR Comoros OR Congo OR ‘Costa Rica’ OR ‘Cote d'Ivoire’ OR ‘Ivory Coast’ OR ‘Cuba’ OR ‘Cuban’ OR ‘Djibouti’ OR ‘Dominica’ OR ‘Ecuador’ OR ‘Egypt’ OR ‘El Salvador’ OR ‘Eritrea’ OR ‘Ethiopia’ OR ‘Fiji’ OR ‘Gabon’ OR ‘Gambia’ OR ‘Georgia’ OR ‘Ghana’ OR ‘Grenada’ OR ‘Guatemala’ OR ‘Guinea’ OR ‘Guyana’ OR ‘Haiti’ OR ‘Honduras’ OR ‘India’ OR ‘Indian’ OR ‘Indonesia’ OR ‘Iran’ OR ‘Iraq’ OR ‘Jamaica’ OR ‘Jordan’ OR ‘Kazakh’ OR ‘Kenya’ OR ‘Kiribati’ OR ‘People's Republic of Korea’ OR ‘North Korea’ OR ‘Kosovo’ OR ‘Kosovar’ OR ‘Kyrgyz’ OR ‘Lao’ OR ‘Laos’ OR ‘Laotian’ OR ‘Lebanon’ OR ‘Lebanese’ OR ‘Lesotho’ OR ‘Liberia’ OR ‘Libya’ OR ‘Macedonia’ OR ‘Madagascar’ OR ‘Malawi’ OR ‘Malaysia’ OR ‘Maldives’ OR ‘Mali’ OR ‘Marshall Island’ OR ‘Mauritania’ OR ‘Mexico’ [MeSH] OR ‘Mexico’ OR ‘Mexican’ OR ‘Micronesia’ OR ‘Moldova’ OR ‘Mongolia’ OR ‘Montenegro’ OR ‘Morocco’ OR ‘Mozambique’ OR ‘Myanmar’ OR ‘Burmese’ OR ‘Burma’ OR ‘Namibia’ OR ‘Nepal’ OR ‘Nicaragua’ OR ‘Niger’ OR ‘Niue’ OR ‘Pakistan’ OR ‘Paraguay’ OR ‘Peru’ OR ‘Philippine’ OR ‘Rwanda’ OR ‘Sao Tome’ OR ‘Principe’ OR ‘Senegal’ OR ‘Serbia’ OR ‘Sierra Leone’ OR ‘Solomon Island’ OR Somalia OR ‘South Africa’ OR ‘Sri Lanka’ OR ‘St Lucia’ OR ‘Saint Lucia’ OR ‘St Vincent’ OR ‘Saint Vincent’ OR ‘Grenade’ OR ‘Sudan’ OR ‘Suriname’ OR ‘Swaziland’ OR ‘Eswatini’ OR ‘Syria’ OR ‘Tajik’ OR ‘Tanzania’ OR ‘Zanzibar’ OR ‘Thai’ OR ‘Timor’ OR ‘Togo’ OR ‘Tonga’ OR ‘Tunisia’ OR ‘Turkey’ OR ‘Turkish’ OR ‘Turkmen’ OR ‘Tuvalu’ OR ‘Uganda’ OR ‘Ukraine’ OR ‘Uzbek’ OR ‘Vanuatu’ OR ‘Venezuela’ OR ‘Vietnam’ OR ‘Vietnam’ OR ‘West Bank’ OR ‘Gaza’ OR ‘Palestine’ OR ‘Yemen’ OR ‘Zambia’ OR ‘Zimbabwe’ OR ‘Western Sahara’ OR ‘Argentina’ OR ‘Russia’ |

**Supplementary Table S2. Characteristics of the included studies providing vitamin supplementation to pregnant and/or lactating women**

| Author, Year, Country | Intervention target | Enrolled sample size | Intervention | Control | Outcomes | Assessment time points | Key findings |
| --- | --- | --- | --- | --- | --- | --- | --- |
| Water-soluble vitamins | | | | | | | |
| Vitamin B-1 | | | | | | | |
| Gallant 2021,  Cambodia [50] | Lactating women | 335 | Daily dose of 1.2, 2.4, or 10 mg of thiamine (vitamin B-1) daily from 2 through 24 weeks postpartum | 0 mg dose of thiamine daily | Milk thiamine, and maternal and infant ThDP | B-1 at 4, 12 and 24 weeks and ThDP at 24 weeks | Milk thiamine concentrations significantly higher in all interventions groups compared with the placebo group |
| Vitamin B-2 | | | | | | | |
| Bates 1983,  Gambia [27] | Lactating women | 60 | Lactating women who had infants between 1- and 18-months during recruitment received daily 2 mg vitamin B-2 for 12 weeks | Placebo | Milk vitamin B-2 concentrations | 2 days before supplementation and 6 times during supplementation | Compared to placebo, milk vitamin B-2 levels increased significantly in intervention group |
| Vitamin B-9 | | | | | | | |
| Batu 1976,  Burma [28] | Pregnant women | 96 | Daily 60 mg elemental iron twice, 5 mg folic acid twice, OR combination of both from 22 to 25 week of the pregnancy till full-term | Placebo | Maternal serum folate concentrations | Baseline (22-25 weeks of pregnancy), near delivery, 4-7 weeks postpartum | Serum folate levels fell in the groups not receiving the hematinic |
| Christian 2006,  Nepal [35] | Pregnant women | 1165 | Daily supplementation throughout pregnancy with: folic acid (400 µg folate), iron folic acid (60g and 400 µg folate), daily folic acid, iron and zinc (30 mg folate), OR multiple micronutrient supplement containing folic acid, iron, zinc and 11 other micronutrients | Daily micronutrient supplement with 1000 µg RE vitamin A and vitamin A alone | Maternal serum folate, B-12, B-6, riboflavin, retinol, α-tocopherol, γ- tocopherol, 25(OH) D concentrations | First and the third trimester | Multiple micronutrient supplementation decreased the prevalence of serum riboflavin, vitamin B-6, vitamin B-12, folate, and vitamin D deficiencies |
| Dijkuizen 2004,  Indonesia [42] | Pregnant women | 170 | Daily IFA with β-carotene (4.5 mg), zinc (30 mg), OR both during pregnancy until delivery; pregnant women recruited before 20-week gestational age | Daily placebo plus iron (30 mg) and folic acid (0.4 mg) | Maternal and infant serum retinol concentrations | 6 months postpartum | Addition of both ß-carotene and zinc to IFA during pregnancy improved the vitamin A status of mothers and their infants |
| Elmacioglu 2010,  Turkey [47] | Pregnant women | 80 | Daily 100 mg Ferro III hydroxide polymaltose complex plus iron pharmaceutical with 0.35 mg folic acid during pregnancy until delivery; pregnant women recruited at 16-24 weeks of their pregnancy | Iron rich diet | Maternal serum B-12 and folate concentrations | Between 16 – 23 weeks, 24 – 28 weeks, 29 – 33 weeks, 34 – 38 weeks, and 39 – 42 weeks of pregnancy | Vitamin B-12 levels were significantly higher in intervention group compared with control group |
| Eneroth 2010,  Bangladesh [48] | Pregnant women | 1066 | Daily 30 OR 60 mg iron and 400 µg folic acid from 14 weeks of gestation to 3 months of postpartum | Daily multiple micronutrients with 30 mg iron and 400 µg folic acid | Infant serum retinol, B-12 and folate concentrations | 6 months of age | Maternal multiple micronutrient supplementation had a beneficial effect on vitamin B-12 status in infancy |
| Ziaei 2016,  Bangladesh [105] | Pregnant women | 871 | Daily 30 OR 60 mg iron and 400 µg folic acid from 14 weeks of gestation to 3 months of postpartum | Daily multiple micronutrient supplements with 30 mg iron and 400 µg folic acid | Maternal serum concentrations of B-12, and folate | 14 and 30 weeks of pregnancy | At week 30 of pregnancy, women in the multiple micronutrient supplements group had higher geometric mean concentrations of vitamin B-12 than women in the Fe60F group |
| Vitamin B-12 | | | | | | | |
| D'Souza 2021,  India [45] | Pregnant women | 266 | Daily vitamin B-12 (2 µg) with or without multiple micronutrients, from preconception until delivery | Placebo | Maternal and cord micronutrients (B-12, Holo-Tc, B-2, and B-6) | At 28 weeks of gestation and delivery | B12 supplemented groups had had higher B-12 levels at pregnancy (28 weeks of gestation), and were reflected in higher cord blood holo-TC levels compared to the placebo group |
| Duggan 2014,  India [46] | Pregnant women | 366 | Daily oral supplementation with vitamin B-12 (50 μg) pregnancy (<14 week of gestation) through 6 weeks postpartum | Placebo | Maternal serum and milk B-12 concentrations and infant B-12 serum concentrations | 1, 2 and 3 trimesters; 6 weeks, and 3 and 6 months postpartum; and, 6 weeks after birth respectively | Oral supplementation of women with vitamin B-12 throughout pregnancy and early lactation significantly increased vitamin B-12 status of mothers and infants soon after delivery, but disappeared by 3 and 6 months postpartum |
| Siddiqua 2016,  Bangladesh [88] | Pregnant women | 68 | Daily 250 μg vitamin B-12 during pregnancy  and 3 months postpartum; women recruited at an early stage of pregnancy (11–14 weeks) | Placebo | Maternal and infant serum B-12, milk, and cord B-12 concentrations | At delivery and 3 months postpartum | Daily supplementation with B12 during pregnancy and lactation substantially improved maternal, infant and milk B12 status |
| Complex B vitamins | | | | | | | |
| Li 2021,  China [61] | Pregnant women | 331 | Daily 60 mg iron OR daily B-complex containing 2 mg of vitamin B-1, 2 mg of vitamin B-2, 2 mg of vitamin B-6, 2 μg of vitamin B-12, 5 mg of vitamin B-5, and 15 mg of vitamin B-3 during pregnancy; pregnant women recruited at < 20 weeks of gestation | No Supplements | Infant serum folate and vitamin B-12 | At delivery | Maternal vitamin B complex supplementation during pregnancy associated with better neonatal vitamin B-12 status |
| Vitamin C | | | | | | | |
| Casanueva 2005,  Mexico [33] | Pregnant women | 126 | Daily 100 mg vitamin C during pregnancy; pregnant women recruited in their 20^th^ week of gestation | Placebo | Maternal serum vitamin C concentrations | At 20, 24, 28, 32 and 36 weeks of gestation | Mean serum vitamin C concentrations decreased significantly throughout the  pregnancy in both groups and no significant differences between groups |
| Fat-soluble vitamins | | | | | | | |
| Vitamin A | | | | | | | |
| Andreto 2012,  Brazil [20] | Lactating women | 312 | 200,000 IU vitamin A capsule to all women; 10 days after delivery: intervention group 200,000 IU of vitamin A | 200000 IU vitamin A and placebo | Maternal serum retinol | Baseline, 2, 4, and 6 months after baseline | No difference was found between the two groups in serum maternal retinol concentration at 2, 4 and 6 months |
| Ayah 2007,  Kenya [23] | Lactating women-infant pairs | 564 | Maternal vitamin A 400,000 IU at <24 hours postpartum and infant vitamin A 400,000 IU at 14 weeks | Placebo | Maternal and infant serum retinol, and milk retinol | 36 weeks of gestation, and 14 and 26 weeks postpartum | No difference in maternal and infant serum retinol between groups; milk retinol higher in vitamin A group |
| Bahl, 2002,  Ghana, India, & Peru [24]^*^ | Lactating women-infant pairs | 631 | A single dose of 60 mg vitamin A to mothers at 18–42 days postpartum and 7.5 mg to infants three times, i.e., at 6, 10 and 14 weeks of age | Placebo | Milk and infant serum retinol | Infants aged 6 weeks, and 6 and 9 months | No effect of supplementation at 9 months; beneficial effect of supplementation in milk retinol and infants’ vitamin A status varied by site |
| Basu 2003,  India [26] | Lactating women | 300 | A single dose of retinol (209 µmol) soon after delivery | No intervention | Retinol level in milk and infant serum | Monthly follow-ups for 6 months | Post supplementation, milk retinol remained higher for 4 months and infants’ serum retinol for 5 months in intervention group |
| Bezerra 2010,  Brazil [29] | Lactating women | 143 | A single retinyl palmitate dose of 200,000 IU OR a double dose of 200,000 IU 24 hours apart; women recruited within 16 hours postpartum | No intervention | Retinol content of colostrum and mature milk | Colostrum milk and milk after 4 weeks | Post supplementation, no difference in retinol in colostrum and milk at 4 weeks postpartum; retinol content differed in mature milk and two intervention groups and control |
| Bhaskaram 1998,  India [30] | Lactating women | 100 | A single dose of 200,000 IU vitamin A orally within 24 hours after delivery | Placebo | Infant serum retinol & milk retinol | Serum retinol from cord blood and 6 weeks; milk retinol at 3, 10, 30, 45 & 90 days | Milk retinol levels higher in the intervention group up to 45-90 days of lactation; for majority of the infant’s serum retinol levels improved significantly by 6 weeks irrespective of the maternal supplementation status |
| DosSantos 2013,  Brazil [44] | Lactating women | 276 | A single dose of 200,000 IU vitamin A capsule to all women; 10 days after delivery: intervention group 200,000 IU of vitamin A | 200000 IU vitamin A + placebo | Infant serum retinol concentration | At 2, 4 and 6 months | Maternal double megadose supplement had no additional effect on the serum retinol levels of the children |
| Grilo 2016,  Brazil [52] | Lactating women | 88 | A single dose 200,000 IU of retinyl palmitate after the first colostrum collection on the first day after delivery | No intervention | Concentration of retinol and a-tocopherol in milk | 0 hour, 24 hours and 30-day milk; blood sample after delivery | Maternal supplementation with high doses of vitamin A increased the colostrum level of it but reduced the bioavailability of a-tocopherol |
| Martines 1998,  Ghana, India, & Peru [64]^*^ | Lactating women and infant pairs | 9424 | A single dose of 200,000 IU vitamin A to mothers (21–42 days postpartum in Ghana and 18–28 days postpartum in India and Peru) and three doses of three doses of 25,000 IU vitamin A to infants at 4, 10 and 14 weeks | Placebo | Infant serum vitamin A concentration | At first dose, 6 months, 9 months and 12 months visits | A small decrease in vitamin A deficiency in the intervention  group compared with controls at 6 months but not at 9 and 12 months |
| Martins 2010,  Brazil [65] | Lactating women | 66 | A single dose of 200,000 IU of vitamin A between 20 and 30 postpartum days | Placebo | Maternal serum and milk retinol concentration and infant retinol concentration | Before supplementation and 3 months after delivery | Supplementation had a positive impact on maternal vitamin A status but no effect on infant status |
| Muslimatun 2001,  Indonesia [69] | Pregnant women | 366 | Weekly iron (120 mg) and folic acid (500 mg) with or without vitamin A (4,800 retinol equivalents) during pregnancy; women recruited at 16–20-week pregnancy | National IFA program (120 mg iron and 500 mg folic acid) | Maternal serum retinol concentration | Baseline and near term | Serum retinol concentrations maintained in the weekly vitamin A and iron group, but decreased in the other two groups |
| Muslimatun 2001a,  Indonesia [70] | Pregnant women | 243 | Weekly iron (120 mg) and folic acid (500 mg) with or without vitamin A (4,800 retinol equivalents) during pregnancy; women recruited at 16–20-week pregnancy | National IFA program (120 mg iron and 500 mg folic acid) | Milk retinol concentration | Transitional milk (4-7 days postpartum) and mature milk (3 months postpartum); maternal serum retinol at 4 months postpartum | Compared with the weekly iron group, the weekly vitamin A and iron group had a greater concentration of retinol in transitional milk and in mature milk but serum retinol concentrations at 4 months postpartum did not differ significantly |
| Rice 1999,  Bangladesh [78] | Lactating women | 221 | A single dose of 200,000 IU vitamin A followed by daily placebos OR daily doses of b-carotene (7.8 mg) until 9 months of postpartum; women recruited at 1-3-week postpartum | Placebo | Maternal and infant serum retinol, milk vitamin A concentrations | Maternal outcomes: 0.5, 3, 6 and 9 months; infant outcome at 6 months of age | No intervention was sufficient to correct the underlying subclinical vitamin A deficiency in the women or bring their infants into adequate vitamin A status |
| Roy 1997,  Bangladesh [83]^*^ | Lactating women | 50 | A single oral dose of 209 µmol of vitamin A at delivery | No intervention | Maternal serum and milk retinol concentration | Prior supplementation, 24 hours, 1, 3, 6 and 9 months after supplementation | Vitamin A supplementation increased milk retinol concentration for at least six months |
| Schmidt 2001,  Indonesia [85] | Pregnant women | 366 | Weekly iron (120 mg) and folic acid (500 mg) with or without vitamin A (4,800 retinol equivalents) during pregnancy; women recruited at 16–20-week gestation | National IFA program (120 mg iron and 500 mg folic acid) | Maternal and infant serum retinol concentrations | 4 months postpartum | Infants of mothers supplemented with vitamin A plus Fe had higher serum retinol concentrations than infants of mothers supplemented with Fe alone, but the proportion of infants with serum retinol deficiency was >70% in all groups |
| Stoltzfus 1993,  Indonesia [91] | Lactating women | 153 | A single dose of 312 µmol of vitamin A as retinyl palmitate at 1-3 weeks of postpartum | Placebo | Maternal and infant serum and milk retinol concentrations | Monthly between 0.5 to 8 months | High dose vitamin A supplementation of lactating mothers improved the vitamin A status of both mother and infant |
| Tanumihardjo 2002,  Indonesia [93] | Pregnant women | 27 | Daily 8.4 µmol (8,000 IU) vitamin A as retinyl palmitate with an iron placebo (A), 1.07 mmol (60 mg) ferrous sulfate with a vitamin A placebo (Fe) and vitamin A plus iron (A + Fe) for 8 weeks; women recruited in the second or early third  trimester | Placebo | Maternal serum retinol concentration | Pre- and post-intervention | Maternal vitamin A status improved in the combination of vitamin A and iron group than by either nutrient alone group |
| Tchum 2006,  Ghana [94] | Lactating women | 168 | 400,000 IU vitamin A in 2 divided doses 24 hours apart 7–10 days after delivery | 200 000 IU as a single dose plus a placebo 24 hours apart | Maternal serum retinol concentration | Pre- and post-intervention | Maternal serum retinol concentrations did not differ in the intervention groups |
| Tomiya 2017,  Brazil [95] | Lactating women | 210 | A single dose 200 000 IU vitamin A at delivery and after 8-10 days postpartum | 200 000 IU as a single dose at delivery | Milk retinol concentration | Colostrum, 2 and 4 months | No significant difference between retinol concentrations in milk between intervention groups |
| Turner 2013,  Bangladesh [97] | Lactating women | 131 | White-fleshed sweet potatoes (WFSPs) with a vitamin A supplement (0.5 mg/d) for 3 weeks; women recruited when they were *>/=2* months into lactation | Placebo and WFSPs, orange-fleshed sweet potatoes, or tangerines | Maternal serum retinol, and milk retinyl equivalent, and milk alpha and gamma tocopherol | Pre- and post-intervention | Vitamin A capsules increased serum and milk vitamin A concentrations |
| Vinutha 2000,  India [100] | Lactating women | 109 | A single dose of vitamin A of 200000 IU within 48 hours of delivery | No intervention | Maternal and infant serum retinol, milk retinol and cord blood retinol concentrations | 3 months postpartum/age; at delivery | Postpartum maternal vitamin A supplementation had a beneficial impact on the infant serum retinol and the milk retinol |
| West 2011,  Bangladesh [102] | Pregnant women | 59666  (5% for blood assessment) | Weekly 7000 µg of retinol equivalents as retinyl palmitate OR 42 mg of all-trans beta carotene from the first trimester of pregnancy through 12 weeks | Placebo | Maternal serum retinol | 1^st^ and 3^rd^ trimester and 12 weeks postpartum | Vitamin A compared with either placebo or beta carotene supplementation increased serum retinol concentrations by end of study |
| Yamini 2001,  Nepal [103] | Pregnant women | 1431 | Weekly 7,000 µg of retinol equivalents as retinyl palmitate OR 42 mg of all-trans beta carotene for 3 weeks; 4 months median gestational age of recruited women | Placebo | Maternal serum retinol, and alpha and gamma tocopherol | At 4 months of gestation and 4 months postpartum | Compared to placebo, In the vitamin A group, serum retinol was higher during pregnancy, and retinol and gamma-tocopherol higher at postpartum |
| Zvandasara 2006,  Zimbabwe [106] | Lactating women | 14110 | A single dose of 400,000 IU vitamin A at less than 96 hours postpartum | Placebo | Maternal serum retinol concentration among HIV negative and positive women | Baseline and 6 weeks postpartum | No significant differences in maternal serum retinol concentration between study groups |
| Vitamin D | | | | | | | |
| Assemi 2015,  Iran [21] | Pregnant women | 46 | Weekly 400IU Vitamin D3 with multi-minerals (calcium, magnesium, and zinc) for 9 weeks | Placebo | Maternal serum vitamin D concentrations | Baseline and 9 weeks after intervention | Compared to the placebo, consumption of multi mineral‑Vitamin D supplements resulted in increased levels of vitamin D |
| Assemi 2016,  Iran [22] | Pregnant women | 42 | Weekly 500 mg calcium carbonate plus 200 IU vitamin D3 for 9 weeks; women recruited at 27 weeks of gestation | Placebo | Maternal serum vitamin D concentrations | Baseline and 9 weeks after intervention | Post supplementation, intervention group had higher level of vitamin D compared to the placebo |
| Bailey 2014,  Bangladesh [25] | Pregnant women | 71 | Weekly 35,000 IU vitamin D until delivery; women recruited in their third trimester of pregnancy (26 to b30 weeks gestation) | Placebo | Maternal, cord, and infant serum vitamin D concentrations | At delivery and 3-28 weeks of age | Vitamin D supplementation substantially  increased mean 25(OH)D3 in maternal and cord specimens relative  to placebo |
| Bugrul 2013,  Turkey [32] | Lactating women and their infants | 96 | Daily 400 IU vitamin D3 to mothers and infants for 6 months; women recruited within 1 month after delivery | No intervention to mothers and daily 400 IU vitamin D3 to infants | Maternal and infant serum vitamin D concentrations | 1 and 6 months of postpartum | At the end of the study, vitamin D levels of mothers and their infants were similar in both groups |
| Chandy 2016,  India [34] | Lactating women and their infants | 230 | 3000 µg/month (120,000 IU) oral vitamin D_3_ for mothers for 9 months OR 10 µg/d (400 IU) for infants; mothers received oral vitamin D_3_ 3000 μg (two sachets of 1500 μg each) within 7 d of delivery, and then 3000 μg at 1·5, 2·5 and 3·5 months on routine immunization visits | Sun exposure | Maternal and infant serum vitamin D concentrations | At 3.5 months | Monthly maternal supplementation with vitamin D along with sun exposure superior to sun exposure alone in maintaining normal infant 25(OH)D at 3·5 months |
| Dabbaghmanesh 2019,  Iran [37] | Pregnant women | 120 | Daily 2000 IU vitamin D_3_ (two 1000 IU pills) from 24-28 weeks of pregnancy until delivery | Placebo | Maternal serum vitamin 25 (OH) D concentrations | Baseline and at birth | At birth, changes in maternal serum level of 25-hydroxyvitamin D significantly higher in the intervention group than control group |
| Dimitris 2016,  Bangladesh [39] | Pregnant women | 65 | Weekly 35,000 IU vitamin D_3_ until delivery; pregnant women recruited at 26–29 weeks gestation | Placebo | Maternal serum vitamin 25 (OH) D concentrations | Baseline and near delivery | Serum D3 remained significantly elevated throughout the week following weekly doses of 35,000 IU D3 in pregnant women |
| Diogenes 2013,  Brazil [41] | Pregnant adolescents | 56 | Daily 600 mg calcium and 200 IU vitamin D_3_ from 26 weeks of pregnancy until delivery | Placebo | Maternal serum vitamin D concentrations | Baseline, 5 and 20 weeks postpartum | In comparison with the placebo, 25(OH)D higher postpartum in the supplemented group |
| Enkhmaa 2019,  Mongolia [49] | Pregnant women | 119 | Daily 300 mg of calcium and 600, 2,000, OR 4,000 IU vitamin D from 12-16 weeks of pregnancy until delivery | N/A | Maternal serum and cord vitamin D concentrations | Baseline; 22 weeks; 36-40 weeks and at delivery respectively | Daily supplementation of 4000 IU during pregnancy improved maternal and neonatal 25(OH)D concentrations than 2000 IU. Daily 600 IU supplements insufficient to prevent vitamin D deficiency |
| Gupta 2018,  India [53]^*^ | Pregnant women | 243 | Daily 600, 1,000, 2,000, OR 4,000 IU vitamin D plus 1000 mg of elemental calcium in two doses until delivery; pregnant women recruited at 12–16 weeks gestation | N/A | Maternal serum and cord vitamin D concentrations | Baseline, 24-28 weeks and at delivery | Supplementation of vitamin D in  mothers improved vitamin D status of newborns |
| Harrington 2014,  Bangladesh [54] | Pregnant women | 160 | Weekly 35,000 IU vitamin D3 until delivery; from 26–29 weeks gestation | Placebo | Maternal serum and cord vitamin 25 (OH) D concentrations | Delivery | Supplementation of vitamin D in  mothers improved vitamin D status of mothers and newborns |
| Hossain 2014,  Pakistan [56] ^*^ | Pregnant women | 200 | Daily 4,000 IU vitamin D3 until delivery; started at 20 weeks gestation | Routine care | Maternal serum vitamin 25 (OH) D concentrations | Delivery | Supplementation  improved maternal vitamin D status |
| Karamali 2015,  Iran [58] | Pregnant women | 60 | Fortnightly 50,000 IU vitamin D3 until delivery; from 20–32 weeks gestation | Placebo | Maternal serum vitamin D concentrations | Baseline and 12 weeks after the intervention | Cholecalciferol supplements  significantly increased maternal serum 25-hydroxyvitamin D concentrations compared with the placebo |
| Mir 2016,  India [66] | Pregnant women | 100 | Daily 1,000 OR 2,000 IU vs monthly 30,000 OR 60,000 IU vitamin D3 until delivery; pregnant women recruited during and up to 16 weeks gestation | N/A | Maternal serum vitamin D3 concentrations | Pre and post supplementation | Vitamin D supplementation with 2000 IU/day or 60,000 IU/month effective and safe in achieving Vitamin D sufficiency in pregnant women |
| Motamed 2019,  Iran [67] | Pregnant women | 84 | Daily 1,000 OR 2,000 IU vitamin D until delivery; pregnant women recruited at *+/<* 12 weeks gestation | N/A | Maternal and cord serum concentration of vitamin 25(OH)D | Pre and post supplementation | Supplementation with 2000 IU/d  vitamin D3 from the first trimester of pregnancy more effective than 1000 IU/d in increasing 25(OH)D levels |
| Motamed 2020,  Iran [68]^*^ | Pregnant women | 84 | Daily 1,000 OR 2,000 IU vitamin D until delivery; pregnant women recruited at the first trimester of pregnancy | N/A | Maternal and cord serum concentration of vitamin 25(OH)D | Pre and post supplementation | Supplementation with 2000 IU/d vitamin D as compared with 1000 IU/d more effective in increasing vitamin D status |
| Mutlu 2014,  Turkey [71] | Pregnant women | 91 | Daily 600, 1,200 OR 2,000 IU vitamin D until delivery regardless of gestational age at recruitment | N/A | Maternal and infant serum concentration of vitamin 25(OH)D | Baseline and 3 mons | Women and their infants in 2000 IU vitamin D supplementation group had higher vitamin D sufficiency than other two groups |
| Naik 2017,  India [72] | Lactating women | 130 | Daily 60,000 IU vitamin D over 10 days in early postpartum period | Placebo | Maternal and infant serum concentration of vitamin 25(OH)D | 6 months postpartum | Infant serum 25(OH)D levels significantly increased at 6 months of age when their mothers supplemented with 60,000 IU/d of vitamin D3, in comparison to placebo group |
| Nausheen 2021,  Pakistan [73] | Pregnant women | 350 | Daily 2,000 OR 4,000 IU vitamin D until delivery; supplementation started between 12- and 16-weeks gestation | Daily 400 IU vitamin D | Maternal and infant serum concentration of vitamin 25(OH)D | Baseline and prior to delivery | Vitamin D supplementation of 4000 IU/d more effective in reducing vitamin D deficiency among pregnant women and in improving serum 25(OH)D levels in mothers and their infants compared with 2000 IU/d and 400 IU/d |
| Niramitmahapanya 2017,  Thailand [74] | Lactating women | 68 | Daily 1,800 vitamin D3 for 6 weeks; started after delivery | Placebo | Maternal serum and milk 25 (OH)D levels | 6 weeks postpartum | Vitamin D supplementation during lactation increased maternal 25(OH)D levels, compared with placebo |
| Ramot 2022,  India [76] | Lactating women | 220 | Monthly 12,000 OR 120,000 IU vitamin D for 12 months; supplementation started within one month of delivery | N/A | Maternal and infant serum 25(OH)D levels | Baseline and 12 months | Bolus vitamin D supplementation dose more efficacious in improving maternal and infant vitamin D status at 12 months, as compared to 12,000 IU/m |
| Raqib 2014,  Bangladesh [77] | Pregnant women | 129 | Weekly 35,000 IU vitamin D_3_ until delivery; starting from 26 weeks of gestation up to delivery | Placebo | Maternal and cord serum concentration of vitamin 25(OH)D | Baseline and delivery | Maternal 25(OH)D concentration in the intervention group significantly  increased at delivery compared with the placebo group |
| Roth 2013,  Bangladesh [79] | Pregnant women | 44 | 70,000 IU once + 35,000 IU/week vitamin D until delivery; pregnant women recruited at 27–30 weeks gestation | Weekly 14000 IU vitamin D | Maternal and cord serum concentration of vitamin 25(OH)D | Baseline and 10 week/70 days | Maternal vitamin D levels increased in initial dose of 70,000 IU and 35,000 IU/week vitamin D3 group than weekly 14000 IU |
| Roth 2013a,  Bangladesh [80]^*^ | Pregnant women | 160 | Weekly 35,000 IU vitamin D until delivery; pregnant women recruited at 26–29 weeks gestation | Placebo | Maternal and cord serum concentration of vitamin 25(OH)D | Baseline and 10 weeks of supplementation | Antenatal vitamin D3 supplementation significantly raised maternal and  cord serum 25(OH)D concentrations above 50 nmol/L, relative to placebo group |
| Roth 2018,  Bangladesh [81] | Pregnant and lactating women | 1298 | Weekly 4,200, 16,800 OR 28,000 to pregnant women IU OR weekly 28,000 IU vitamin D during pregnancy to postpartum; supplementation at 17–24 weeks gestation | Placebo | Maternal, cord and infant serum concentration of vitamin 25(OH)D | Baseline, delivery, 3 and 6 months postpartum | Vitamin D had dose-dependent effects on concentrations of maternal, cord blood, and infant 25-hydroxyvitamin D |
| Rothberg 1982,  South Africa [82] | Lactating women | 40 | Daily 500 OR 1,000 IU vitamin D for 6 weeks following delivery | Placebo | Maternal and infant serum concentration of vitamin 25(OH)D | 4 days of delivery and 6 weeks | After six weeks, intervention group mothers and their infants had higher levels of 25-hydroxyvitamin D than mothers receiving placebo |
| Sablok 2015,  India [84] | Pregnant women | 165 | Depending on vitamin D levels; supplementation started at 14–20 weeks gestation | No intervention | Maternal and infant serum concentration of vitamin 25(OH)D | Baseline and at delivery | Intervention group mothers and their infants had higher levels of 25-hydroxyvitamin D than mothers in no intervention group |
| Soheilykhah 2013,  Iran [89] | Pregnant women | 120 | Daily 200 IU vs 50,000 IU/every two weeks or 50,000 IU monthly vitamin D until delivery; supplementation started at 12 weeks gestation | N/A | Maternal serum concentration of vitamin 25(OH)D | Baseline and at delivery | An appropriate dose of vitamin D supplementation during pregnancy could be 4000 IU vitamin D daily or 50 000 IU every 2 weeks |
| Trivedi 2020,  India [96] | Lactating women | 132 | Vitamin D_3_ 60,000 IU between 24 and 48 hours postpartum and at 6, 10, and 14 weeks amounting to 240,000 IU of vitamin D3 | Vitamin D3 60,000 IU between 24 and 48 hours postpartum and Placebo | Infant serum concentration of vitamin 25(OH)D | At recruitment and 6 months age | Infant serum 25(OH)D concentration increased significantly when mothers supplemented with 240,000 IU of vitamin D3 during lactation in comparison with the infants of mothers in placebo group |
| Vaziri 2016,  Iran [98] | Pregnant women | 153 | Daily 2,000 IU vitamin D until delivery; supplementation started at 26–28 weeks gestation | Placebo | Maternal serum concentration of vitamin 25(OH)D | Baseline and at delivery | Intervention group had significantly higher vitamin D levels at delivery relative to placebo group |
| Vaziri 2016a,  Iran [99] | Pregnant women | 153 | Daily 2,000 IU vitamin D until delivery; supplementation started at 26–28 weeks gestation | Placebo | Maternal serum concentration of vitamin 25(OH)D | Baseline and at delivery | Intervention group had significantly higher vitamin D levels at delivery relative to placebo group |
| Vitamins A & D | | | | | | | |
| Ding 2021,  China [40] | Lactating women | 294 | Daily oral vitamin A and D drops (one soft capsule of 1800 IU vitamin A and 600 IU vitamin D2) for 2 months; lactation women recruited at 30–45 days postpartum | Placebo | Maternal serum and milk retinol levels | Pre- and post-supplementation | After the 2-month intervention, maternal serum retinol concentrations increased in the supplementation group with no change in the control group |
| Vitamin E | | | | | | | |
| Clemente 2015,  Brazil [36] | Lactating women | 109 | A single dose of 400 IU of RRR-α-tocopherol or 400 IU of all-rac- α - tocopherol following the first milk collection; approximately 12 hours postpartum | No supplementation | Maternal colostrum α tocopherol | Before and 24 hours after supplementation | Supplementation with both forms of a-TOH increased vitamin E concentrations in colostrum; however, the natural form more efficient in increasing the levels |
| De Sousa Reboucas 2019,  Brazil [38] | Lactating women | 79 | A single dose of 800 IU of RRR-alpha-tocopherol after the first collection of milk; women recruited between 30 and 90 days after delivery | No supplementation | Maternal serum and milk vitamin E concentrations | Before and 24 hours after supplementation | In the supplemented group, the alpha-tocopherol content in serum and milk increased after supplementation |
| Lira 2017,  Brazil [62] | Lactating women | 50 | A single dose of 400 IU of RRR-α-tocopherol immediately after the first collection of serum | No supplementation | Maternal serum vitamin E concentrations | Day 1, 20, 30 and 60 after delivery | A single vitamin E supplement did not change the mean serum level of a-tocopherol in postpartum women |
| PiresMedeiros 2016,  Brazil [75] | Lactating women | 81 | A single dose of 400 IU of RRR-α-tocopherol after the collection of milk after delivery | No supplementation | Maternal serum vitamin E concentrations | 24 hours after the first collection, as well as 7 and 30 d after delivery | Maternal supplementation with 400 IU of RRR-α-tocopherol increased the  vitamin E concentrations of the colostrum and transitional milk, but not of the mature milk |
| Multiple micronutrient supplements (MMS) | | | | | | | |
| Bhutta 2009,  Pakistan [31] | Pregnant women | 2378 | Daily MMS (UNIMMAP formulation) during pregnancy; pregnant women recruited with a confirmed pregnancy at less than  16 weeks gestation | Daily 60 mg iron and 400 µg folic acid | Maternal serum concentrations of vitamin A | Pre- and post-supplementation | No significant differences in the maternal serum concentrations of vitamin A between two intervention groups |
| Garcia-Guerra 2009,  Mexico [51] | Pregnant women | 507 | Daily MMS containing 100 to 150% of the recommended dietary allowance for zinc, iron, vitamins A, C, B-6, folate, and several other micronutrients from approximately 9 weeks of pregnancy until delivery | Daily 60 mg iron and 400 µg folic acid | Maternal serum concentrations of retinol, and folate, cord blood retinol, and milk retinol concentrations | At delivery and 1 month postpartum | MMS during pregnancy did not improve vitamin A status and some indication that folate status may be improved compared to Fe only |
| Haskell 2021,  Malawi [55] | Pregnant women | 318 | Daily MMS (800 μg retinol equivalent (RE) /capsule), or SQ-LNS (800 μg RE/20g) during pregnancy and the first 6 months postpartum OR SQ-LNS (a 20-g sachet that contained 800 μg RE plus the same 17 micronutrients as the MMS capsule and 4 additional minerals: calcium, phosphorus, potassium, magnesium); pregnant women recruited at ≤20 weeks of gestation | Daily 60 mg iron and 400 µg folic acid | Maternal and infant serum retinol and inflammation corrected serum retinol, and milk retinol | Maternal outcomes: 36 weeks of gestation and 6 months of postpartum; infant outcomes: 6 and 18 months of age | Supplementation had no effect on maternal or infant vitamin A status |
| Haskell 2021,  Ghana [55] | Pregnant women | 292 | Daily MMS (800 μg retinol equivalent (RE)/capsule), or SQ-LNS (800 μg RE/20g) during pregnancy and the first 6 months postpartum OR SQ-LNS (a 20-g sachet that contained 800 μg RE plus the same 17 micronutrients as the MMS capsule and 4 additional minerals: calcium, phosphorus, potassium, magnesium); pregnant women recruited at ≤20 weeks of gestation | Daily 60 mg iron and 400 µg folic acid | Infant serum retinol and inflammation corrected serum retinol | 6 and 18 months of age | Supplementation had no effect on infant vitamin A status |
| Jones 2020,  Gambia [57] | Pregnant women | 863 | Daily MMS including 10 μg cholecalciferol, protein energy as lipid-based supplement OR multiple micronutrient supplement plus protein energy; from < 20 weeks gestation until delivery | Daily iron and folic acid tablets as per Gambian Government guidelines | Maternal serum 25(OH)D3 | Early and late pregnancy | MMS increased maternal vitamin D status |
| Khambalia 2006,  Mexico [59]^*^ | Lactating women | 83 | Daily MMS containing a-tocopherol (30 mg), vitamin C (500 mg), thiamin (10 mg), riboflavin (10 mg), niacinamide (100 mg), vitamin B-6 (5mg), vitamin B12 (12mg), biotin(45mg), pantothenic acid (20mg), and folic acid (400 mg) with OR without iron (18 mg) for 6 months; women recruited at the first clinic visit after childbirth | N/A | Maternal serum and milk folate | 22-, 82- and 138-day of postpartum | No significant differences in the proportion of women with folate deficiency by treatment group |
| Klevor 2016,  Ghana [60] | Pregnant women | 1320 | Daily MMS providing 18 micronutrients, including 800 mg retinol equivalents of vitamin A OR lipid-based supplement with the same nutrients as the MMS group, plus 4 minerals and macronutrients supplemented from < 20 weeks gestation 6 months of postpartum | Daily supply of iron and folic acid (IFA) only during pregnancy and a calcium placebo tablet during the first 6 months of postpartum | Milk retinol and fat concentrations | At 6 months postpartum | Daily consumption of approximately the recommended intake of vitamin A did not increase milk retinol concentrations |
| Lweno 2020,  Tanzania [63] | Pregnant women | 491 | Daily multivitamins containing 50 µg vitamin B12 during pregnancy (from 12–27 weeks gestation) and in the postpartum period | Placebo | Milk B-12 concentration | At 6 weeks postpartum 7 months postpartum | Multivitamin supplements did not significantly reduce the odds of inadequate vitamin B12 in milk |
| Schulze 2019,  Bangladesh [86] | Pregnant women | 1526 | MMS recommended by UNICEF with 27 mg iron and 600 μg folic acid from early to late pregnancy | Daily 27 mg iron and 600 μg folic acid | Maternal serum concentrations of folate, vitamins A, α-tocopherol, B-12, E, and D | Pre-supplementation and 32 weeks of post-supplementation | MMS improved micronutrient status, although deficiencies persisted |
| Schulze 2020,  Bangladesh [87] | Pregnant women | 333 | MMS recommended by UNICEF with 27 mg iron and 600 μg folic acid from early to late pregnancy | Daily 27 mg iron and 600 μg folic acid | Cord blood serum concentrations of folate, vitamins A, α-tocopherol, B-12, E, and D | At delivery | MMS increased newborn 25(OH)D, while maternal and newborn folate, vitamins B-12, D, and E, biomarkers were positively related |
| Taghizadeh 2014,  Iran [92] | Pregnant women | 48 | 10 multivitamin supplements for 20 weeks during pregnancy; pregnant women recruited in their second and third trimester | 13 multivitamin and mineral supplements | Maternal and infant serum concentrations of vitamin D | Baseline and 20 weeks of supplementation, and at birth respectively | No significant difference in maternal and infant vitamin D status between the intervention groups |
| Wang 2023,  Tanzania [101] | Pregnant women (supplemented till postpartum) | 357 | Daily multivitamins containing 50 µg vitamin B12 during pregnancy (from 12–27 weeks gestation) and in the postpartum period | Placebo | Milk B-12 concentration | At 6 weeks postpartum 7 months postpartum | Prenatal supplementation increased milk vitamin B-12 concentration at 6 weeks postpartum, but not at 7 months postpartum |
| Lipid-based nutrient supplement (LNS) | | | | | | | |
| Donohue 2020,  Guatemala [43]^*^ | Lactating women | 28 | Bolus 30-g dose of LNS OR 3 × 10-g doses of LNS (Divided); LNS contained vitamins A, B-1, B-2, B-3, B-5, B-6, B-12, folate, C, D, E, K and multiple minerals at 4–6 months postpartum | No LNS | Milk concentrations and infant intake from milk of B-vitamins (B-1, B-2, B-3, B-6, B-12) | 8 hours after supplementation | Maternal supplementation with LNS as a bolus or divided dose similarly effective at increasing milk riboflavin, thiamin, and pyridoxal and infant intakes, whereas only the bolus dose increased cobalamin, niacin was unaffected in 8 h. |
| Haskell 2021,  Malawi [55] | Pregnant women | 210 | Daily MMS (800 μg retinol equivalent (RE)/capsule), or 3) SQ-LNS (800 μg RE/20g) during pregnancy and the first 6 months pf postpartum OR SQ-LNS (a 20-g sachet that contained 800 μg RE plus the same 17 micronutrients as the MMS capsule and 4 additional minerals: calcium, phosphorus, potassium, magnesium); pregnant women recruited at ≤20 weeks of gestation | Daily 60 mg iron and 400 µg folic acid | Maternal and infant serum retinol and inflammation corrected serum retinol, and milk retinol | Maternal outcomes: 36 weeks of gestation and 6 months of postpartum; infant outcomes: 6 and 18 months of age | SQ-LNS had no effect on vitamin A status of mothers or children |
| Haskell 2021,  Ghana [55] | Pregnant women | 292 | Daily MMS (800 μg retinol equivalent (RE)/capsule), or 3) SQ-LNS (800 μg RE/20g) during pregnancy and the first 6 months postpartum OR SQ-LNS (a 20-g sachet that contained 800 μg RE plus the same 17 micronutrients as the MMS capsule and 4 additional minerals: calcium, phosphorus, potassium, magnesium); pregnant women recruited at ≤20 weeks of gestation | Daily 60 mg iron and 400 µg folic acid | Infant serum retinol and inflammation corrected serum retinol | 6 and 18 months of age | SQ-LNS had no effect on vitamin A status of mothers or children |
| Klevor 2016,  Ghana [60] | Pregnant women | 1320 | Daily MMS providing 18 micronutrients, including 800 mg retinol equivalents of vitamin A OR lipid-based supplement with the same nutrients as the MMS group, plus 4 minerals and macronutrients supplemented from < 20 weeks gestation till 6 months of postpartum | Daily supply of iron and folic acid (IFA) only during pregnancy and a calcium placebo tablet during the first 6 months of postpartum | Milk retinol and fat concentrations | At 6 months postpartum | No significant differences in milk retinol concentrations between study groups |
| Stewart 2020,  Madagascar [90]^*^ | Pregnant and lactating women | 387 | T1 plus LNS for children aged 6-8 months (T2); T2 plus LNS for pregnant/lactating women (T3); OR T1 plus parenting messages (T4)  LNS for women: 40 g/d, providing ∼200 kcal/d, 50% of the iron daily needs, and 1–2 times the recommended daily amount of micronutrients for pregnant women recruited in their second or third trimester | Routine program with monthly growth monitoring and nutrition education (T0) OR Routine program with intensive nutrition counseling (T1) | Infant vitamin A serum concentrations | Towards the end of LNS supplementation | No significant differences in infant vitamin A status between study groups |
| Young 2021,  Guatemala, India, and Pakistan [104] | Pregnant women | 200 | Daily lipid-based multiple micronutrient supplements containing vitamin A, B-1, B-2, B-3, B-5, B-6, B-12, C, D2, E, and K along with other micronutrients, for at least 3 months prior to conception; Arm 2 received the daily lipid-based multiple micronutrient supplement beginning at 12–14 weeks pregnancy | No supplementation | Milk concentrations of vitamin B-1, B-2, B-3, B-5, B-6, B-7, and B-12 | At 2-6 weeks of postpartum | Prenatal supplementation for at least 6 months had no impact on milk B-vitamin concentrations at 2-weeks postpartum |

Note: *Studies did not contribute to meta-analyses

**Supplementary Table S3: Risk of bias of the included studies**

| Study | Bias arising for the randomization process | Bias due to deviation from the intended intervention | Bias due to missing outcome data | Bias in the measurement of the outcome | Bias in the selection of the reported results | For cluster randomized trials only: Bias arising from the timing of identification and recruitment of individual participants in relation to timing of randomization | Overall bias |
| --- | --- | --- | --- | --- | --- | --- | --- |
| **Water-soluble vitamins** | | | | | | | |
| Vitamin Bs | | | | | | | |
| Bates, 1983 | Low | Low | High | Low | Some concerns |  | High |
| D'Souza, 2021 | Some concerns | Low | Some concerns | Low | Some concerns |  | High |
| Duggan, 2014 | Low | Low | High | Low | Some concerns |  | High |
| Gallant, 2021 | Low | Low | Low | Some concerns | Low |  | Some concerns |
| Li, 2021 | High | Low | Low | Some concerns | Low | Low | High |
| Siddiqua, 2016 | Low | Low | Some concerns | Some concerns | Some concerns |  | High |
| Batu, 1976 | Some concerns | High | Some concerns | High | High |  | High |
| Christian, 2006 | Low | Low | Some concerns | Low | Low |  | Some concerns |
| Dijkuizen, 2004 | Some concerns | Some concerns | Low | Low | Low |  | High |
| Elmacioglu, 2010 | Some concerns | Low | Low | Low | Low |  | Some concerns |
| Eneroth, 2010 | Low | Low | Some concerns | Low | Low |  | Some concerns |
| Ziaei, 2016 | Low | Low | Some concerns | Low | Low |  | Some concerns |
| **Vitamin C** |  |  |  |  |  |  |  |
| Casanueva, 2005 | Low | Low | Some concerns | Low | Some concerns |  | Some concerns |
| **Fat-soluble vitamins** | | | | | | | |
| **Vitamin A** | | | | | | | |
| Andreto, 2012 | Low | Low | High | Low | Low |  | High |
| Ayah, 2007 | Low | Low | Some concerns | Low | Low |  | Some concerns |
| Bahl, 2002 | Low | Low | Some concerns | Low | Low |  | Some concerns |
| Basu 2003 | High | Low | High | Low | Low |  | High |
| Bezerra, 2010 | Some concerns | Low | Low | Low | Some concerns |  | Some concerns |
| Bhaskaram, 1998 | High | Low | Low | Low | Some concerns |  | Some concerns |
| Dos Santos, 2013 | Low | Low | Some concerns | Low | Low |  | Some concerns |
| Grilo, 2016 | Some concerns | Low | High | Low | Low |  | High |
| Martines, 1998 | Low | Low | Low | Low | Low |  | Low |
| Martins, 2010 | Low | Low | Low | Low | Low |  | Low |
| Muslimatun, 2001 | Some concerns | Some concerns | Some concerns | Low | Low |  | High |
| Muslimatun, 2001a | Some concerns | Some concerns | Some concerns | Low | Low |  | High |
| Rice, 1999 | Low | Low | Some concerns | Low | Low |  | Some concerns |
| Roy, 1997 | High | Some concerns | Low | High | High |  | High |
| Schmidt, 2001 | Some concerns | Some concerns | Some concerns | Low | Low |  | High |
| Stoltzfus, 1993 | Some concerns | Some concerns | Some concerns | Low | Low |  | High |
| Tanumihardjo, 2002 | High | Low | Low | Low | Some concerns |  | High |
| Tchum, 2006 | High | High | High | High | High |  | High |
| Tomiya, 2017 | Low | Low | Some concerns | Low | High |  | High |
| Turner, 2013 | Low | Low | Some concerns | Low | Some concerns |  | Some concerns |
| Vinutha, 2000 | Some concerns | Low | High | Low | High |  | High |
| West, 2011 | Some concerns | Low | High | Low | Low | Low | High |
| Yamini, 2001 | Some concerns | Low | Some concerns | Low | Low |  | Some concerns |
| Zvandasara, 2006 | Some concerns | Some concerns | High | Low | Low |  | High |
| **Vitamin D** | | | | | | | |
| Assemi, 2015 | Some concerns | Low | Some concerns | Low | High |  | High |
| Assemi, 2016 | Some concerns | Low | Some concerns | Low | High |  | High |
| Bailey, 2014 | High | Low | Low | Low | Low |  | High |
| Bugrul, 2013 | High | Low | High | Low | Low |  | High |
| Chandy, 2016 | Low | Low | High | Low | High |  | High |
| Dabbaghmanesh, 2019 | Low | Low | High | Low | High |  | High |
| Dimitris, 2016 | High | Low | Low | Low | Low |  | High |
| Diogenes, 2013 | Some concerns | Some concerns | High | High | Some concerns |  | High |
| Enkhmaa, 2019 | Low | Low | Some concerns | Low | Low |  | Some concerns |
| Gupta, 2018 | Low | Low | High | Low | High |  | High |
| Harrington, 2014 | Low | Low | High | Low | High |  | High |
| Hossain, 2014 | Low | Low | High | High | High |  | High |
| Karamali, 2015 | Low | Low | Low | Low | Low |  | Low |
| Mir, 2016 | Some concerns | Low | High | Low | Low |  | High |
| Motamed, 2019 | Low | Low | High | Low | Low |  | High |
| Motamed, 2020 | Low | Some concerns | Low | Some concerns | Some concerns |  | High |
| Naik, 2017 | Low | Low | High | Low | Low |  | High |
| Nausheen, 2021 | Low | Low | High | Low | Low |  | High |
| Niramitmahapanya, 2017 | Some concerns | Low | Some concerns | Low | Some concerns |  | High |
| Ramot, 2022 | Some concerns | Low | Some concerns | Low | Low |  | High |
| Raqib, 2014 | Some concerns | Some concerns | Low | Some concerns | Some concerns |  | High |
| Roth, 2013 | Some concerns | Some concerns | Low | Some concerns | Some concerns |  | High |
| Roth, 2013a | Low | Low | Low | Low | Low |  | Low |
| Roth, 2018 | Low | Low | Some concerns | Low | Low |  | Some concerns |
| Rothberg, 1982 | High | Low | Low | Low | Low |  | High |
| Sablok 2015 | High | Low | High | Low | High |  | High |
| Soheilykha, 2013 | High | Low | High | High | Low |  | High |
| Trivedi, 2020 | Low | Low | High | Low | Low |  | High |
| Vaziri, 2016 | Low | Some concerns | Low | Some concerns | Some concerns |  | High |
| Vaziri, 2016a | Low | Low | High | Low | High |  | High |
| YesiltepeMutlu, 2014 | High | Low | High | Low | Low |  | High |
| **Vitamin A and D** | | | | | | | |
| Ding, 2021 | Some concerns | Low | Some concerns | Low | Low |  | High |
| **Vitamin E** | | | | | | | |
| Clemente, 2015 | Some concerns | Low | Some concerns | Low | Low |  | High |
| De Sousa Reboucas, 2019 | Some concerns | Low | Some concerns | Low | Low |  | High |
| Lira, 2017 | Some concerns | Low | Some concerns | Low | Low |  | High |
| PiresMedeiros, 2016 | High | Some concerns | Some concerns | High | Some concerns |  | High |
| **Multiple micronutrient supplements (MMS)** | | | | | | | |
| Bhutta, 2009 | Some concerns | Low | Some concerns | Low | Some concerns |  | High |
| Garcia-Guerra, 2009 | High | Some concerns | High | Low | Some concerns |  | High |
| Haskell, 2021 (Malawi) | Some concerns | Some concerns | Some concerns | Low | Some concerns |  | High |
| Haskell, 2021 (Ghana) | Some concerns | Some concerns | Some concerns | Low | Some concerns |  | High |
| Jones, 2020 | Some concerns | Some concerns | Some concerns | High | Some concerns |  | High |
| Khambalia, 2006 | Low | Low | Low | Low | Some concerns |  | Some concerns |
| Klevor, 2016 | Low | Some concerns | Some concerns | Low | Some concerns |  | High |
| Lweno, 2020 | Low | Low | Low | Low | Low |  | Low |
| Schulze, 2019 | Low | Low | Low | Low | Low |  | Low |
| Schulze, 2020 | Low | Low | Low | Low | Low |  | Low |
| Taghizadeh, 2014 | Low | Low | Low | Low | Low |  | Low |
| Wang, 2023 | Some concerns | Low | Low | Low | Low |  | Low |
| **Lipid-based nutrient supplement (LNS)** | | | | | | | |
| Donohue, 2020 | Low | Low | Low | Low | Low |  | Low |
| Haskell, 2021 (Malawi) | Some concerns | Some concerns | Some concerns | Low | Some concerns |  | High |
| Haskell, 2021 (Ghana) | Some concerns | Some concerns | Some concerns | Low | Some concerns |  | High |
| Klevor, 2016 | Low | Some concerns | Some concerns | Low | Some concerns |  | High |
| Stewart, 2020 | Some concerns | Low | Low | Low | Low | Low | Some concerns |
| Young, 2021 | Low | Low | Low | Low | Low |  | Low |

**Supplementary Table S4: Assessment of Certainty of Evidence Using the GRADE Approach**

| Outcome | Sub-group of interventions | Study design | Risk of bias | Inconsistency | Indirectness | Imprecision | Other considerations | Certainty |
| --- | --- | --- | --- | --- | --- | --- | --- | --- |
| **Water-soluble vitamins** | | | | | | | | |
| **Thiamin (B-1) studies** | | | | | | | | |
| Vitamin B-1 level in milk | All studies supplementing B-1 | RCTs | Not serious | Serious | Not serious | Not serious | Publication bias suspected | Very low |
|  | Vitamin B-1 2.4 mg – 2.8 mg dose | RCTs | Not serious | Serious | Not serious | Not serious | Publication bias suspected | Very low |
| **Riboflavin (B-2) studies** | | | | | | | | |
| Vitamin B-2 level in milk | All studies supplementing B-2 | RCTs | Serious | Not serious | Not serious | Not serious | None | Low |
| **Folate (B-9) studies** | | | | | | | | |
| Vitamin B-9 concentration in maternal serum | All studies supplementing folate | RCTs | Serious | Not serious | Not serious | Not serious | Publication bias suspected | Very low |
|  | IFA only | RCTs | Serious | Not serious | Not serious | Not serious | Publication bias suspected | Very low |
|  | 350 ug – 400 ug folate | RCTs | Serious | Not serious | Not serious | Not serious | Publication bias suspected | Very low |
|  | Folate concentration categorized as =/ <6.8umol/L | RCTs | Serious | Not serious | Not serious | Not serious | Publication bias suspected | Very low |
| Vitamin B-9 concentration in cord serum | All studies supplementing folate | RCTs and one CRCT | Serious | Serious | Not serious | Not serious | None | Very low |
| **Cobalamin (B-12) studies** | | | | | | | | |
| Vitamin B-12 concentration in maternal serum | All studies supplementing vitamin B12 | RCTs | Not serious | Not serious | Not serious | Not serious | Publication bias suspected | Very low |
|  | Excluding supplementation from preconception | RCTs | Not serious | Not serious | Not serious | Not serious | Publication bias suspected | Very low |
|  | Vitamin B-12 only | RCTs | Not serious | Not serious | Not serious | Not serious | Publication bias suspected | Very low |
|  | MMS containing vitamin B-12 | RCTs | Not serious | Not serious | Not serious | Not serious | Publication bias suspected | Very low |
|  | 2 – 3 ug of vitamin B-12 | RCTs | Not serious | Not serious | Not serious | Not serious | Publication bias suspected | Very low |
|  | Vitamin B-12 concentration categorized as =/<150 pmol/L | RCTs | Not serious | Not serious | Not serious | Not serious | Publication bias suspected | Very low |
| Vitamin B-12 level in milk | All studies supplementing vitamin B12 | RCTs | Not serious | Not serious | Not serious | Not serious | Publication bias suspected | Very low |
|  | Vitamin B-12 only | RCTs | Serious | Not serious | Not serious | Not serious | Publication bias strongly suspected | Very low |
|  | MMS and/or LNS containing vitamin B-12 | RCTs | Serious | Not serious | Not serious | Not serious | Publication bias suspected | Very low |
|  | Vitamin B-12 in pregnancy only | RCTs | Serious | Not serious | Not serious | Not serious | Publication bias suspected | Very low |
|  | 50 ug vitamin B-12 | RCTs | Serious | Not serious | Not serious | Not serious | Publication bias suspected | Very low |
|  | Vitamin B-12 concentration categorized as =/<310 pmol/L | RCTs | Serious | Not serious | Not serious | Not serious | None | Very low |
| Vitamin B-12 concentration in infant serum | All studies supplementing vitamin B12 | RCTs | Serious | Not serious | Not serious | Not serious | Publication bias suspected | Very low |
|  | Vitamin B-12 concentration categorized as =/<150 pmol/L | RCTs | Serious | Not serious | Not serious | Not serious | Publication bias suspected | Very low |
| Vitamin B-12 concentration in cord serum | All studies supplementing vitamin B-12 | RCTs and one CRCT | Serious | Not serious | Not serious | Not serious | **None** | Very low |
|  | Vitamin B-12 concentration categorized as =/<150 pmol/L | RCTs and one CRCT | Serious | Not serious | Not serious | Not serious | **None** | Very low |
| **Fat-soluble vitamins** | | | | | | | | |
| **Vitamin A studies** | | | | | | | | |
| Vitamin A concentration in maternal serum | All studies supplementing vitamin A | RCTs and one CRCT | Serious | None | None | None | Publication bias suspected | Low |
|  | Vitamin A only | RCTs and one CRCT | Serious | None | None | None | Publication bias suspected | Low |
|  | Retinol or retinyl palmitate only | RCTs and one CRCT | Serious | None | None | None | Publication bias suspected | Very low |
|  | Retinol only | RCTs | Serious | None | None | None | Publication bias suspected | Very low |
|  | Retinyl palmitate only | RCTs and one CRCT | Serious | None | None | None | Publication bias suspected | Very low |
|  | Beta carotene only | RCTs and one CRCT | Serious | None | None | None | Publication bias suspected | Very low |
|  | Vitamin A supplementation only in pregnancy | RCTs | Serious | None | None | None | None | Low |
|  | Vitamin A supplementation only in postpartum | RCTs | Serious | None | None | None | None | Low |
|  | Single dose of Vitamin A (200,000 IU – 400,000 IU) | RCTs | Serious | None | None | None | Publication bias suspected | Very low |
|  | Continuous dose of vitamin A | RCTs and one CRCT | Serious | None | None | None | None | Low |
|  | Vitamin A supplementation compared to placebo | RCTs and one CRCT | Serious | None | None | None | None | Low |
|  | Vitamin A concentration categorized as =/<1.05umol/L | RCTs | Not serious | None | None | None | Publication bias suspected | Low |
|  | Vitamin A concentration categorized as =/<0.7umol/L | RCTs | Not serious | None | None | None | Publication bias suspected | Low |
| Vitamin A level in milk | All studies supplementing vitamin A | RCTs and one CRCT | Not serious | Not serious | Not serious | Not serious | Publication bias suspected | Moderate |
|  | Vitamin A only | RCTs and one CRCT | Serious | Not serious | Not serious | Not serious | None | Low |
|  | Retinyl palmitate only | RCTs and one CRCT | Serious | Not serious | Not serious | Not serious | None | Low |
|  | Vitamin A supplementation only in pregnancy | RCTs and one CRCT | Serious | Not serious | Not serious | Not serious | None | Low |
|  | Vitamin A supplementation only in postpartum | RCTs and one CRCT | Serious | Not serious | Not serious | Not serious | None | Low |
|  | Single dose of Vitamin A (200,000 IU – 400,000 IU) | RCTs | Serious | Not serious | Not serious | Not serious | None | Low |
|  | Vitamin A supplementation compared to placebo | RCTs and one CRCT | Serious | Not serious | Not serious | Not serious | None | Low |
|  | Vitamin A concentration categorized as =/<1.05umol/L | RCTs | Serious | Not serious | Not serious | Not serious | Publication bias suspected | Low |
|  | Vitamin A concentration categorized as =/<0.7umol/L | RCTs | Serious | Not serious | Not serious | Not serious | Publication bias suspected | Low |
| Vitamin A concentration in infant serum | All studies supplementing vitamin A | RCTs | Serious | Not serious | Not serious | Not serious | None | Very low |
|  | Vitamin A only | RCTs | Serious | Not serious | Not serious | Not serious | None | Low |
|  | Retinol or retinyl palmitate only | RCTs | Serious | Not serious | Not serious | Not serious | Publication bias suspected | Low |
|  | Retinyl palmitate only | RCTs | Serious | Not serious | Not serious | Not serious | Publication bias suspected | Low |
|  | Vitamin A supplementation only in pregnancy | RCTs | Serious | None | None | None | Publication bias suspected | Very low |
|  | Vitamin A supplementation only in postpartum | RCTs | Serious | Not serious | Not serious | Not serious | None | Low |
|  | Single dose of Vitamin A (200,000 IU – 400,000 IU) | RCTs | Serious | Not serious | Not serious | Not serious | None | Low |
|  | Vitamin A supplementation compared to placebo | RCTs | Serious | Not serious | Not serious | Not serious | None | Low |
|  | Vitamin A deficiency =/<0.7umol/L | RCTs | Serious | Not serious | Not serious | Not serious | None | Low |
| Vitamin A concentration in cord serum | All studies supplementing vitamin A | RCTs | Serious | Not serious | Not serious | Not serious | Publication bias suspected | Low |
| **Vitamin D studies** | | | | | | | | |
| Vitamin D concentration in maternal serum | All studies supplementing vitamin D | Not serious | Not serious | Serious | Not serious | Not serious | Publication bias suspected | Low |
|  | Vitamin D only | RCTs | Not serious | Serious | Not serious | Not serious | Publication bias suspected | Very low |
|  | Vitamin D supplementation in pregnancy only | RCTs | Not serious | Serious | Not serious | Not serious | Publication bias suspected | Very low |
|  | Vitamin D supplementation in postpartum only | RCTs | Not serious | Serious | Not serious | Not serious | Publication bias suspected | Very low |
|  | Daily dose of =/< 2, 000 IU vitamin D | RCTs | Serious | Serious | Not serious | Not serious | Publication bias suspected | Very low |
|  | Daily dose of >2,000 IU vitamin D | RCTs | Serious | Serious | Not serious | Not serious | Publication bias suspected | Very low |
|  | Vitamin D supplementation compared to placebo | RCTs | Serious | Serious | Not serious | Not serious | Publication bias suspected | Very low |
|  | Vitamin D concentration categorized as =/<50nmol/L | RCTs | Serious | Serious | Not serious | Not serious | Publication bias suspected | Moderate |
| Vitamin D concentration in infant serum | All studies supplementing vitamin D | RCTs | Serious | Serious | Not serious | Not serious | None | Very low |
|  | Vitamin D supplementation in pregnancy only | RCTs | Serious | Serious | Not serious | Not serious | None | Very low |
|  | Vitamin D supplementation in postpartum only | RCTs | Serious | Serious | Not serious | Not serious | None | Very low |
|  | Vitamin D supplementation compared to placebo | RCTs | Serious | Serious | Not serious | Not serious | None | Very low |
|  | Vitamin D concentration categorized as =/<50nmol/L | RCTs | Serious | Not serious | Not serious | Not serious | Publication bias suspected | Very low |
| Vitamin D concentration in cord serum | All studies supplementing vitamin D | RCTs | Serious | Not serious | Not serious | Not serious | Publication bias suspected | Very low |
|  | Vitamin D only |  | Serious | Not serious | Not serious | Not serious | Publication bias suspected | Very low |
|  | Vitamin D supplementation compared to placebo | RCTs | Serious | Not serious | Not serious | Not serious | Publication bias suspected | Very low |
|  | Vitamin D concentration categorized as =<50nmol/L | RCTs | Not serious | Not serious | Not serious | Not serious | None | Low |
| **Vitamin E studies** | | | | | | | | |
| Alpha tocopherol concentration in maternal serum | All studies supplementing vitamin E | RCTs | Serious | Not serious | Not serious | Not serious | None | Very low |
|  | Vitamin E supplementation in pregnancy only | RCTs | Serious | Not serious | Not serious | Not serious | None | Very low |
|  | Vitamin E supplementation in postpartum only | RCTs | Serious | Not serious | Not serious | Not serious | None | Very low |
| Gamma Tocopherol concentration in maternal serum | All studies supplementing vitamin E | RCTs | Serious | Not serious | Not serious | Not serious | None | Very low |
| Alpha tocopherol level in milk | All studies supplementing Vitamin E | RCTs | Serious | Not serious | Not serious | Not serious | None | Very low |
|  | Vitamin E only | RCTs | Serious | Not serious | Not serious | Not serious | None | Very low |

CRCT: Clustered Randomized Controlled Trial; IU: International Unit; RCT: Randomized Controlled Trial
